# Agent-based modeling of the central amygdala and pain using cell-type specific physiological parameters

**DOI:** 10.1371/journal.pcbi.1009097

**Published:** 2021-06-08

**Authors:** Rachael Miller Neilan, Gabrielle Majetic, Mauricio Gil-Silva, Anisha P. Adke, Yarimar Carrasquillo, Benedict J. Kolber

**Affiliations:** 1 Department of Mathematics and Computer Science, Duquesne University, Pittsburgh, Pennsylvania, United States of America; 2 Department of Biological Sciences, Duquesne University, Pittsburgh, Pennsylvania, United States of America; 3 Department of Engineering, Duquesne University, Pittsburgh, Pennsylvania, United States of America; 4 Department of Neuroscience and Center for Advanced Pain Studies, University of Texas at Dallas, Richardson, Texas, United States of America; 5 National Center for Complementary and Integrative Health, National Institutes of Health, Bethesda, Maryland, United States of America; National Research Council, ITALY

## Abstract

The amygdala is a brain area involved in emotional regulation and pain. Over the course of the last 20 years, multiple researchers have studied sensory and motor connections within the amygdala in trying to understand the ultimate role of this structure in pain perception and descending control of pain. A number of investigators have been using cell-type specific manipulations to probe the underlying circuitry of the amygdala. As data have accumulated in this research space, we recognized a critical need for a single framework to integrate these data and evaluate emergent system-level responses. In this manuscript, we present an agent-based computational model of two distinct inhibitory neuron populations in the amygdala, those that express protein kinase C delta (PKC*δ*) and those that express somatostatin (SOM). We utilized a network of neural links to simulate connectivity and the transmission of inhibitory signals between neurons. Type-specific parameters describing the response of these neurons to noxious stimuli were estimated from published physiological and immunological data as well as our own wet-lab experiments. The model outputs an abstract measure of pain, which is calculated in terms of the cumulative pro-nociceptive and anti-nociceptive activity across neurons in both hemispheres of the amygdala. Results demonstrate the ability of the model to produce changes in pain that are consistent with published studies and highlight the importance of several model parameters. In particular, we found that the relative proportion of PKC*δ* and SOM neurons within each hemisphere is a key parameter in predicting pain and we explored model predictions for three possible values of this parameter. We compared model predictions of pain to data from our earlier behavioral studies and found areas of similarity as well as distinctions between the data sets. These differences, in particular, suggest a number of wet-lab experiments that could be done in the future.

## Author summary

In this manuscript, we present a computational modeling approach to understand and predict pain output from a part of the brain, the amygdala, involved in stress adaptation, emotional regulation, and pain. Over the last several years, a variety of groups have begun to dissect the specific cells that are responsible for the impact of amygdala activation on pain, which can include both increases and decreases in pain and pain-like output in animal models. It is helpful and necessary to use computational models to develop a framework to understand the amygdala as these wet lab techniques add to the complexity of our understanding of the brain structure. The model presented here was based on our recent published physiology experiments along with multiple examples of expression data. This model can be used to design future wet-lab experiments and can continue to be refined to help us evaluate the impact of the amygdala and similar limbic system structures in pain and other disease. Here we present the first computational model for amygdala signaling that includes physiological and histological properties of neurons and allows dynamic simulation of nociceptive signal propagation through the network.

## Introduction

Pain serves as a critical survival signal for organisms to avoid tissue damage and to prevent exacerbating existing injuries. In the context of chronic pain, the adaptative nature of pain is absent as the pain persists beyond healing and/or occurs in the absence of an identifiable injury. While the peripheral nervous system no doubt plays a critical role in pain chronification and maintenance of chronic pain, the central nervous system from the spinal cord to the brainstem and brain likely contribute heavily to the cognitive and emotional toll of disease in patients. As such, there has been intense interest in identifying the brainstem and brain areas that are engaged during both acute and chronic pain states.

Brain areas related to pain can be roughly split into those contributing to sensory-discriminative, cognitive, and emotional/adaptive responses to noxious sensory cues. The main sensory pathway for spinal nociceptive information is the spinothalamic tract which ends in the thalamus, whereby information is sent to areas such as the somatosensory cortex, anterior cingulate cortex, and prefrontal cortical areas. Other pathways, such as the spinoparabrachial tract, relay nociceptive signals more directly to sub-cortical structures such as the amygdala through the parabrachial nucleus (PBN) in the brainstem. Finally, multiple brain areas contribute to descending control of pain through brainstem areas such as the periaqueductal gray (PAG) and rostral ventral medulla (RVM).

To understand the emotional and motivational aspects of pain and injury, many researchers have focused on the amygdala, an area involved in stress adaptation and fear conditioning. The classical view of the amygdala posits that highly processed information comes in from cortical structures to the lateral and basolateral amygdala (BLA) before final integration and efferent output from the central nucleus of the amygdala (CeA). In fact, bi-directional connections between the BLA and the prefrontal cortex appear to play a central role in emotional decision-making that is disrupted after injury[1]. However, the CeA also receives nociceptive information from the PBN to a subnucleus (the lateral capsular division or CeC). This “pain nucleus” of the amygdala then sends signals to the descending pain modulatory system.

Over 15 years ago, we discovered that the right and left CeA had differential impact in the context of peripheral inflammatory pain in mice[2,3,4]. The right CeA seemed to be activated to a greater degree after peripheral injection of formalin compared to the left CeA. Furthermore, inhibition of this activation (or artificial activation in the absence of injury) in the right CeA only influenced pain-like behavior. These findings were replicated in rats[5], found to be related to metabotropic glutamate receptor signaling[4,6], and were relevant in the context of neuropathic pain. Interestingly, after neuropathic injury the left amygdala was initially hyperactive, but eventually the right amygdala picked up the dominant role as seen in acute models[7]. Overall, these results fit into a larger data set including data from human chronic pain conditions that shows hemispheric differences in the amygdala in pain[8]. We have found that in the context of visceral bladder stimulation, the left amygdala appeared to have a tonic analgesic activity at baseline while the right amygdala had little role in uninjured animals[9]. Nonetheless, optogenetic activation of the right amygdala could drive bladder-pain-like physiological changes and optogenetic activation of the left amygdala after bladder injury could reduce pain-like changes. Expanding on these data, we published a preliminary agent-based model (ABM) of the CeA that was calibrated using pilot extracellular recordings from the left and right CeA after bladder injury[10]. Although only preliminary, this ABM reproduced changes in pain similar to those observed in wet-lab experiments during optogenetic inhibition of the left and right amygdala.

As technology has improved, there has been a strong push in the last 10+ years to further dissect the internal circuitry of the CeA[11,12,13,14]. Although most cells in the CeA are GABAergic inhibitory neurons, the nucleus is heterogeneous and sub-type specificity plays a critical role in the ultimate output and impact of CeA activity on behavior[15]. Most recently, we found opposing roles of two largely non-overlapping populations of right CeA neurons in the cuff model of neuropathic pain where a small piece of tubing is placed along the left sciatic nerve[16]. We found that neurons expressing protein kinase C-delta (PKC*δ*) had a pro-nociceptive role in amygdala pain output. These PKC*δ* neurons could be driven in naïve mice to induce peripheral mechanical hypersensitivity, and inhibition of these neurons after injury reduced pain-like behavior. In contrast, somatostatin (SOM) positive CeA neurons had an opposing impact on pain such that inhibition of these neurons in naïve animals increased pain. Overall, these opposing roles of PKC*δ* and SOM in the right amygdala suggest that the left/right lateralization data may be more complicated than initially thought. For example, one could imagine that different proportions of PKC*δ* and SOM neurons in the right and left might lead to an overall pronociceptive role for the right amygdala and an overall antinociceptive role for the left amygdala. There is currently controversy in the literature regarding proportions of PKC*δ*:SOM neurons and the potential for left and right differences[17,18]. In this manuscript, we present a computational model to explore this controversy and, more broadly, to be used by neuroscientists as a tool to quickly perform experiments prior to committing years of effort in the laboratory.

We describe here an agent-based approach to modeling the PKC*δ* and SOM neurons in the left and right amygdala and their activity before, during, and after injury. Agent-based models (ABMs) are used increasingly in all disciplines to study emergent features of complex systems governed by the actions and interactions of individual agents[19,20,21,22]. In the field of neuroscience, ABMs have been used to describe the dynamics and connectedness of individual neurons (or regions of the brain) and to evaluate emergent system-level behavior that could not have been determined using the individual components alone[23,24]. In our ABM, each PKC*δ* and SOM neuron is represented by an individual agent and assigned type-specific properties based on data obtained from wet-laboratory experiments. Neural connectivity is established using a network of directed links through which agents send inhibitory signals to one another. During a simulation of the ABM, individual-level neuron properties are updated each time step in response to a noxious stimulus and a system-level measure of pain is outputted.

Whenever possible, we used data from laboratory experiments to parameterize and validate our computational model. In the Methods section below, we start with a summary of the laboratory experiments that informed the design and analysis of the model. Subsequently, we provide a detailed description of the model using a standard protocol for describing ABMs. All files and code needed to simulate the model are available via a public repository (see Section 2.8). Step-by-step instructions for running the model, as well as images of the model’s graphical user interface (GUI), are provided in the Supporting Information (**S1 Text and S1 Fig**). In the Results section, we demonstrate the model’s ability to predict pain for a variety of scenarios and parameter values and highlight a current controversy in the literature surrounding a key model parameter, the expression of PKC*δ*:SOM.

## Methods

### Ethics statement

Data from unpublished experiments were performed in accordance with the guidelines of the National Institutes of Health (NIH) and were approved by the Animal Care and Use Committee of the National Institute of Neurological Disorders and Stroke (NINDS) and the National Institute on Deafness and other Communication Disorders (NIDCD). All steps were taken to reduce animal suffering in experimentation including careful monitoring of animal weights and signs of distress. All animals were humanely euthanized. Ethics statements from published data can be found in original citations.

### 1 Laboratory experiments

There are four primary experiments that were used to build our CeA ABM. These experiments include published and unpublished data and are indicated as such in subheadings below. When data are unpublished, the methods are presented below in detail and data are described in the Results section.

### 1.1 Electrophysiological Measurement of PKC*δ* and SOM Neurons in the CeA

#### 1.1.1 Electrophysiological data of PKCδ vs SOM neurons in brain slices from control and injured mice (published data)

**Fig 1:**
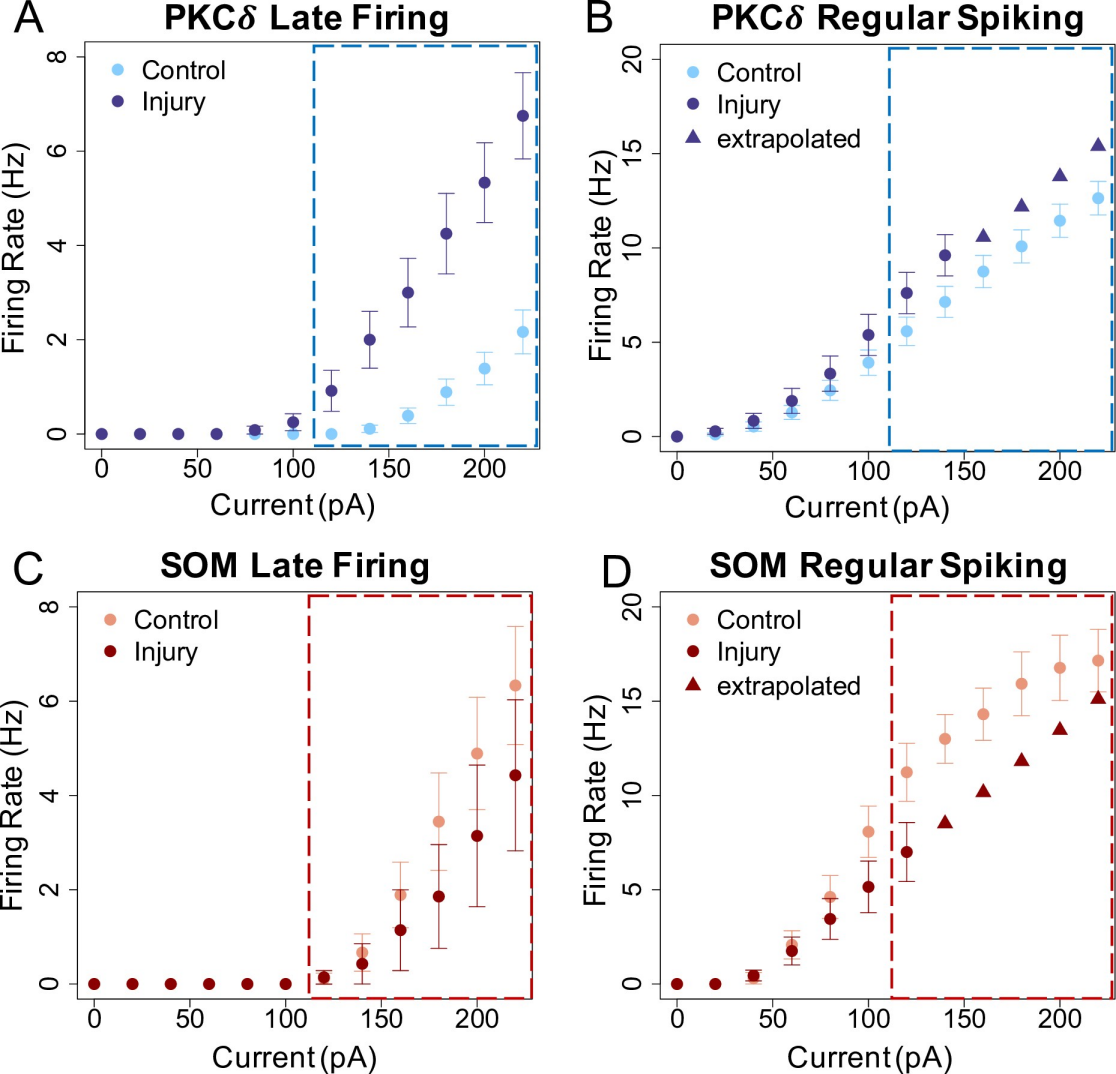
Slice physiology recorded PKC*δ* and SOM positive neurons in control and injured mice. The firing rate at high current injection of PKC*δ* positive neurons is higher after injury for both (A) late firing and (B) regular spiking neurons compared to control slices. In contrast, the firing rate at high current injection of SOM positive neurons is lower after injury for both (C) late firing and (D) regular spiking neurons. Triangles represent extrapolated data in B and D for post-injury regular spiking neurons. Boxed values represent firing rates used in model simulations. Some of these raw data have been previously reported[16,26].

We performed *ex-vivo* slice physiology of amygdala brain slices from sham, naïve, and Sciatic cuff implantation (“cuff model”) mice 1–2 weeks after surgery. For the purpose of calibrating the ABM, we combined published data from the sham and naïve mice to a single “control” group. The cuff model was completed as described[16,25]. Briefly, a 2mm-long-piece of PE-20 tubing is split along its length and wrapped around the left sciatic nerve of male mice. To identify PKC*δ* vs SOM neurons, we utilized two reporter lines of mice. For PKC*δ* studies, *Prkcd*-cre mice (GENSAT-founder line 011559-UCD) were mated with Ai9(RCL-tdT) reporter mice (Jackson Labs, 007909) to visualize and record from PKC*δ* neurons. For SOM studies, heterozygous *Sst*-Cre mice (Jackson Labs, 018973) were bred with Ai9 reporter mice. Whole cell current-clamp recordings were obtained from slices. Single and repetitive action potential firings were elicited from resting membrane potentials in response to brief (5 ms) and prolonged (500 ms) depolarizing current injections of variable amplitudes, respectively. Spontaneously active neurons were recorded for 5 minutes, gap-free, in the cell-attached configuration, and 1-minute gap-free after break-in. Neurons were found to be spontaneously active or were regular spiking (RS) or late firing (LF). Data showing the firing rates of RS and LF neurons are presented in **Fig 1**. The firing rates of spontaneous SOM neurons were statistically significantly higher compared to spontaneous PKC*δ* neurons as recently reported[26]. We found spontaneous neurons to fire at a constant rate of 2.838 Hz (PKC*δ*) or 4.887 Hz (SOM).

#### 1.1.2 Electrophysiological measurement of PKCδ and SOM neurons in the CeA (unpublished data extrapolation)

At higher current injections in the above published data[16], we observed depolarization block in both PKC*δ* and SOM RS neurons. Therefore, in order to use the full data set of neuronal firing in the ABM, we extrapolated the firing rates at higher currents as depicted in the triangles seen in **Fig 1**. Extrapolated values were obtained by fitting a linear model to the positive firing rates at lower currents.

#### 1.1.3 Proportion of spontaneously active, RS, or LF PKCδ and SOM neurons in the CeA (published and unpublished data)

**Fig 2:**
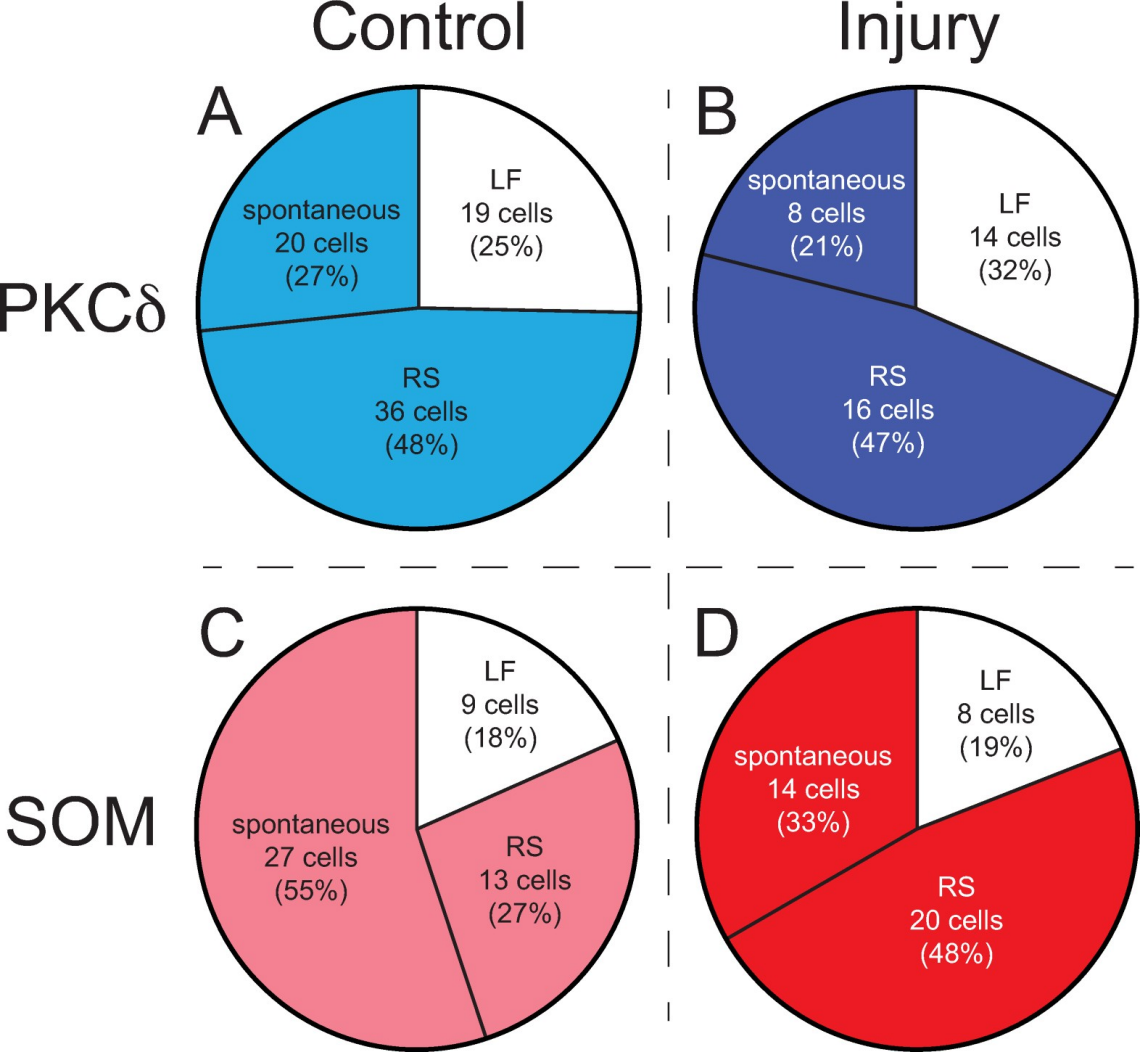
Proportions of PKC*δ* and SOM late firing, regular spiking, and spontaneous neurons after injury. In PKC*δ* neurons, the proportions of late firing (LF), regular spiking (RS), and spontaneous cells do not change from control **(A)** to injury **(B)**. In SOM neurons, the proportions do appear to change. In control slices, there are more spontaneous cells **(C)** compared to slices from injured mice which exhibit less spontaneous cells and more RS cells **(D)**. Data in **(A)** and **(C)** have been previously published[26].

We evaluated the proportions of both PKC*δ* and SOM neurons that were of differing firing types. Proportions of firing type in control conditions were recently published in Adke et al, 2020[26] (**Fig 2A and 2C**). New analyses of electrophysiological data from Wilson et al, 2019[16] are presented here for neurons following injury (**Fig 2B and 2D**) where we show the number of cell-type specific neurons that fall into the three classes of firing rates as described above.

### 1.2 Evaluation of the Connections of PKC*δ* and SOM Positive Neurons in the CeA

#### 1.2.1 Networking of CeA neurons (published data)

The CeA contains GABAergic interneurons and projection neurons. To account for networking between neurons, we utilized specific published data to approximate the proportion of connections that would exist between different types of cells (i.e. PKC*δ* and SOM). The axons of neurons can make synapses at thousands of locations on a dendrite[27]. A biophysically realistic computational model of this system would be challenging to create. Therefore it is common for models to simulate connectivity by making each cell behave as an integrate-and-fire neuron[28]. By focusing only on primary dendrites, the complexity of the connections is reduced. Morphological data of CeC neurons show that they have on average 3.5 primary dendrites that each branch multiple times as reported by others[29] [30] [31] and consistent with our recent work[26]. Limiting our number of connections to these primary dendrites and axons simplifies our model while also allowing for interpretations of how connections change the network properties. An important concern given this decision is whether cell type determines the level of connectivity of a cell. Previous studies have found that firing types of cells showed differing averages of primary dendrites, meaning that our assumption to use the same number of connections for all cells may not be accurate[32]. However, we recently directly compared PKC*δ* and SOM morphology and found no statistically significant difference between the number of primary dendrites in the CeC[26]. In our simulations, we explored model output for four different networks of primary connections (e.g. primary dendrites or primary axonal branches): (1) no connectivity, (2) exactly 1 input and 1 output per neuron, (3) at most 3 inputs and 3 outputs per neuron, and (4) at most 5 inputs and 5 outputs per neuron regardless of whether the cell was PKC*δ* or SOM.

#### 1.2.2 Cell-Type connections between CeA neurons (published data)

Beyond the number of connections between cells, we were also interested in modeling the signature of the connections. Since both PKC*δ* and SOM in the CeA can make intra-CeA connections and projections from the CeA, it is important to consider the various types of connections made. For this part of the model, we utilized a published data set from Hunt et al 2017[17]. In those experiments, the authors utilized a combination of transgenic expression markers and immunohistochemistry to determine the frequency of PKC*δ*-to-PKC*δ*, PKC*δ*-to-SOM, PKC*δ*-to-other, SOM-to-SOM, SOM-to-PKC*δ*, and SOM-to-other. To extract these data from the paper, we identified all classified connected neurons from the manuscript (n = 20). Of these connections, 55% were SOM-to-SOM, 20% were PKC*δ*-to-PKC*δ*, 10% were PKC*δ*-to-SOM, and 15% SOM-to-PKC*δ*.

### 1.3 Evaluation of the number of PKC*δ* and SOM positive neurons in the left and right CeA

As mentioned in the introduction, a major factor in the relationship between pronociceptive PKC*δ* neurons and antinociceptive SOM neurons could be the relative quantity of these neurons in the amygdala on both the right and left sides. To estimate values for these critical model parameters, we used one published data set, completed our own experiment, and considered a default scenario in which the two cell types exist in equal proportions.

#### 1.3.1 Number of CeA PKCδ and SOM neurons (published data)

This published data came once again from Hunt et al 2017[17] described above. In that publication, immunohistochemistry was utilized to label CeA neurons in adult male mice as being PKC*δ* or SOM positive. The proportion of PKC*δ* compared to SOM cells was reported to not be significantly different between the right and left CeA so reported numbers were pooled. They found that of the neurons counted 48% ± 5% were PKC*δ* and 38% ± 3% were SOM. A small pool of neurons showing overlap (1.5 ± 0.5%) were ignored in our analysis as were those neurons not expressing either marker. Therefore, for the purpose of simulating our model based on these data, we assumed 56% were PKC*δ* positive and 44% were SOM positive. This model is referred to as the “60:40” model in the results section.

#### 1.3.2 Number of CeA PKCδ and SOM neurons (unpublished data)

In contrast, to the data from Hunt et al 2017, another published data set[18] has shown almost double the number of SOM neurons compared to PKC*δ* in the amygdala (side of brain was not specified). To clarify this discrepancy in the literature and further evaluate the potential for differential expression of PKC*δ* or SOM neurons in the right and left CeA, we performed our own expression study using heterozygous *Sst*-Cre mice bred with Ai9 tdTomato reporter mice to visualize and count SOM neurons. At the end of each experiment, mice were deeply anesthetized with 1.25% Avertin anesthesia (2,2,2-tribromoethanol and tert-amyl alcohol in 0.9% NaCl; 0.025 ml/g body weight), then perfused transcardially with 0.9% NaCl (37°C), followed by 100 mL of ice-cold 4% paraformaldehyde (PFA). The brain was dissected, cut in half to separate the right and left hemispheres, and postfixed in 4% PFA overnight at 4°C. After cryoprotection in 30% sucrose for 48 h, coronal sections (30 μm) were obtained using a freezing sliding microtome and stored in 0.1 M Phosphate Buffered Saline (PBS), pH 7.4 containing 0.01% sodium azide (Sigma) at 4°C until immunostaining. Sections were rinsed in PBS, incubated in 0.1% Triton X-100 in PBS for 10 minutes at room temperature and blocked in 5% normal goat serum (NGS) (Vector Labs, Burlingame, CA) with 0.1% Triton X-100, 0.05% Tween-20 and 1% bovine serum albumin (BSA) for 30 minutes at room temperature. Sections were then incubated for 72 h at 4°C in mouse anti-PKCδ primary antibody (1:1000, BD Biosciences, 610397). Sections were then rinsed in PBS and incubated in Alexa Fluor 647-conjugated goat anti-mouse (1:100, Invitrogen, A21235) in 1.5% NGS blocking solution with 0.1% Triton X-100, 0.05% Tween 20 and 1% BSA, protected from light, for 2 h at room temperature. Sections were then rinsed in PBS, mounted on positively charged glass slides, air-dried and coverslips were placed using Fluoromount-G (SouthernBiotech). High magnification z-stack images were collected at 0.9 μm steps with a 20x objective using a Nikon A1R laser scanning confocal microscope. The anatomical limits of the CeA were delineated using the mouse brain atlas[33]. An experimenter blind to the side of hemisphere counted the cells positive for PKCδ and SOM (i.e. tdTomato) using the Nikon Elements software. 5–8 sections from each animal (a total of 4 animals) were mounted anterior to posterior covering -0.58 to -1.94 mm Bregma. The total number of cells on each section was counted and averaged across the anterior to posterior extent of the CeA separating the left and right amygdala. Note that we recently published[26] the anterior to posterior cell numbers for the right CeA only data.

### 1.4 Behavioral evaluation of PKC*δ* and SOM neurons in the CeA in a model of neuropathic injury (published data)

Finally, we utilized our previously published behavioral data showing the impact of manipulating PKC*δ* neurons and SOM neurons in control and neuropathic injured male mice using the cuff model described above (Methods Section 1.1.1)[16]. Following one week recovery from surgery, mice were tested for thermal and mechanical sensitivity. Most relevant to the present manuscript, cuffed mice exhibit long-lasting mechanical hypersensitivity. Cuff mice were compared to sham operated animals as well as naïve animals. To manipulate PKC*δ* and SOM neurons, we previously utilized inhibitory and excitatory Designer Receptors Exclusively Activated by Designer Drugs (DREADDs). Viral-based cre-recombinase dependent DREADD vectors were injected into the right CeAs of male mice from either *Prkcd*-cre (founder line 011559-UCD) or *Sst*-Cre (Jackson Labs, 018973) transgenic strains. As described in the introduction above and previously reported, we found that PKC*δ* was largely nociceptive and SOM was largely antinociceptive in uninjured and injured mice. For PKC*δ*, this corresponded to “Fig 4G” (right graph) for inhibition of PKC*δ* and “Fig 4H” (right graph) for activation of PKC*δ* from Wilson et al 2019[16]. For SOM, this corresponded to “Fig 5G” (right graph) for inhibition of SOM and “Fig 5H” (right graph) for activation of SOM from Wilson et al 2019[16]. These data were used to validate system-level output generated from our ABM. For comparisons between experimental data and model output, we utilized a standardized mean effect calculation (see Methods Section 2.9.2 below).

### 1.5 Statistics for laboratory experiments

For laboratory experiments, most data was pulled directly from Hunt et al, 2017[17] or Wilson et al, 2019[16]. New statistical analyses were completed with GraphPad Prism (v 8.0) on the expression of PKC*δ* and SOM in the left and right amygdala. 2-way repeated measures ANOVA with Sidak multiple comparisons were used with P<0.05 considered statistically significant for expression analysis.

### 2 Model description

Next we present a summary of the ABM. This section is written in accordance to the Overview, Design concepts, and Details (ODD) protocol[34,35], which is a standard format for describing ABMs.

### 2.1 Purpose and basic principles

The purpose of this ABM is to synthesize individual-level properties of PKC*δ* and SOM neurons observed in different laboratory experiments into a single framework and to assess emergent system-level properties within the CeA. The following basic principles guided the development of the ABM. Details and equations for all model procedures can be found in Section 2.7 Submodels.

#### Individual-level neuron properties

Model agents represent individual neurons and are assigned type-specific properties and behaviors based on data collected from the laboratory experiments described above. Specifically, experimental results from Hunt et al 2017[17], Wilson et al 2019[16] and new experiments were used to estimate the proportion of PKC*δ* and SOM neurons in each hemisphere of the CeA. As mentioned above in Methods Section 1.1.3, the proportions of PKC*δ* and SOM neurons, respectively, that are LF, RS, and spontaneous replicate those observed in our publication Adke et al 2020[26] and with new analysis of data from Wilson et al 2019[16] (**Fig 2**). The firing rate of each PKC*δ* and SOM neurons in the ABM is updated at each time step using probability distributions estimated from data in Wilson et al 2019[16]. These firing rates depend on the stimulation current (pA) and the damage that is accumulated by the neuron in the model.

#### Neural Connectivity

Local connectivity and inhibition of neurons within the CeA plays an important role in pain modulation. Neural connectivity in the ABM is established using a network of directed links through which agents send inhibitory signal to one another. A stochastic algorithm creates a network of unidirectional links at the start of each model simulation using the connectivity rates (i.e. proportion of PKC*δ*-to-PKC*δ*, PKC*δ*-to-SOM, SOM-to-SOM, and SOM-to-PKC*δ* connections) from Hunt et al 2017[17] (Methods Section 1.2.2 above). At each time step, if the sum of all incoming signals for a neuron exceeds a threshold value, the neuron is silenced.

#### Damage Accumulation

Damage accumulation models (also known as damage-repair models) are used to describe the effects of stressors over time on the health of biological systems[36,37,38]. Previously, a pilot damage accumulation model was used to simulate the impact of noxious, external stimulations on the sensitization of excited and inhibited neurons in the CeA[10]. A similar damage accumulation model is used in the present ABM. During periods of noxious stimulation (i.e. injury), neurons accrue “damage” which over time leads to sensitization and behavioral changes consistent with those observed in the laboratory experiments. All PKC*δ* and SOM neurons are assigned a damage level equal to zero at initialization and accrue damage at a randomly assigned rate during periods of stimulation. The amount of damage accrued by a neuron is then used to determine the neuron’s level of sensitization due to injury. For both PKCδ and SOM neurons, sensitization impacts the neuron’s firing rate. Additionally, for SOM neurons only, sensitization can cause some spontaneous neurons to convert to the RS firing type.

#### Emergence

The primary emergent feature of the model is a measure of pain that evolves over time and in response to external stimulation. In the model, pain is measured as the difference between the cumulative firing rates of all pro-nociceptive PKC*δ* neurons and the cumulative firing rates of all anti-nociceptive SOM neurons across the left and right hemispheres of the CeA. During each time step in an ABM simulation, individual neuron properties are updated and a system level measure of pain is outputted.

### 2.2 Entities, state variables, and scale

The model consists of 1640 agents representing neurons within the CeA. Of these, 1600 agents represent individual neurons that express either PKC*δ* or SOM and are responsive to neuropathic injury in animal models, while the remaining 40 agents represent an arbitrary number of “other” neurons within the CeA. The spatial domain is a 40x41 grid with 1640 patches, each marking the location of one agent. The spatial domain is divided into halves representing the left and right hemispheres of the CeA. Directed links between agents represent unidirectional, inhibitory connections between neurons. Each model tick represents one time-step.

Each of the 1600 agents representing individual PKC*δ* or SOM neurons has ten variables (**Table 1**) defining its properties and behavior. During initialization, each neuron (i.e. agent) is assigned a location (*Loc*) indicating whether it is within the left or the right hemisphere of the CeA and a protein-expression type (*Type*) equal to either PKC*δ* or SOM. A neuron’s location and protein-expression type remain constant throughout a simulation. Each neuron has a firing frequency pattern (*Freq*) equal to regular spiking (RS), late firing (LF), or spontaneous (Spont). Each neuron has three variables (*d*, *t*_*L*_, *t*_*S*_) related to its “damage.” The damage variable (*d*) represents the percentage of total damage accumulated by a neuron in response to external stimulation. The rate at which a neuron accumulates damage during stimulation depends on its damage latency period (*t*_*L*_) and sensitivity (*t*_*S*_). Additionally, each neuron has three variables related to its connectivity with other neurons within the CeA. Two of these variables are the neuron’s number of incoming connections (*Num-In-Link*) and number outgoing connections (*Num-Out-Link*). The third variable is the neuron’s inhibition status (*Inhibited*), which is a binary variable indicating whether the neuron is inhibited or not. Lastly, each neuron has a firing rate (*Fr*) describing the frequency in hertz (spikes per second) of the neuron’s action potentials.

**Table 1:** Overview of variables assigned to agents representing individual neurons in the ABM. In the instances where the parameter values were estimated from laboratory data, the appropriate reference is provided.

Variable	Description	Value	Frequency of updates	Reference
*Loc*	Neuron location within CeA	Left or Right	Assigned at initialization	
*Type*	Protein expression type	PKCδ or SOM	Assigned at initialization	50% PKC*δ* /50% SOM (default setting) 40% PKC*δ* /60% SOM [17] 30% PKC*δ* /70% SOM (Fig 6)
*Freq*	Firing frequency	Late Firing (LF), Regular Spiking (RS), or Spontaneous (Spont)	Updated each time step	Figs 1 and 2; [16,26]
*t*_*L*_	Length of damage latency period	integer in [40,80]	Assigned at initialization	
*t*_*S*_	Length of sensitizing period	integer in [50,150]	Assigned at initialization	
*d*	Damage (percent of total damage)	real number in [0,100]	Updated each time step	
*Num-In-Link*	Number of inputs	integer in [0,5]	Assigned and updated at initialization	
*Num-Out-Link*	Number of outputs	integer in [0,5]	Assigned and updated at initialization	
*Inhibited*	Inhibition status	Yes or No	Assigned at initialization and updated each time step	
*Fr*	Firing Rate	non-negative real number	Updated each time step	Fig 1; [16]

The remaining 40 agents labeled as “Other” represent an arbitrary number of neurons in the CeA that do not express either PKC*δ* or SOM. Inclusion of these neurons is consistent with previous studies that have shown that while SOM and PKC*δ* neurons comprise most of the CeA, there is a small percentage of neurons that do not express either PKC*δ* or SOM[17,39]. These “Other” agents are necessary for the creation of the neural network in which some connections transmit signals from either PKC*δ* or SOM neurons to other neurons in the CeA. The “Other” neurons do not send inhibitory signals to PKC*δ* or SOM neurons and do not contribute to pain-related model output. Therefore, the “Other” neurons are not assigned the additional variables in **Table 1**.

Each connection (i.e., directed link) in the neural network has three variables (**Table 2**) describing how inhibitory signals are transmitted between its endpoints. Each connection has a transmitting endpoint (*end*1) equal to the ID of the agent that is the source of the signal and a receiving endpoint (*end*2) equal to the ID of the agent that is the destination of the signal. Each connection has a signal strength (*str*) equal to the firing rate of neuron associated with its transmitting endpoint. When used, the neural network is established during the model’s initialization and does not change during a simulation.

**Table 2:** Overview of variables assigned to each connection between neurons.

Variable	Description	Value	Frequency of updates
*end1*	ID of agent that is sending the inhibitory signal	positive integer	Assigned at initialization
*end2*	ID of agent that is receiving the inhibitory signal	positive integer	Assigned at initialization
*str*	Strength of inhibitory signal transmitted	non-negative real number	Updated each time step

### 2.3 Global variables and input data

Global variables *Max*_*in*_ and *Max*_*out*_ control the maximum number of incoming connections and maximum number of outgoing connections, respectively, an individual neuron can possess. Both *Max*_*in*_ and *Max*_*out*_ are non-negative integer values that must be set by the user prior to the model’s initialization.

The timing, duration, and magnitude of external stimulation (measured in pA) must be input as a file consisting of integer values, ranging from 0 to 220. During initialization, the values in the file are read one at a time and stored as a global vector, *S*, where *S*_*i*_ represents the stimulation (pA) applied during the i^th^ time step. In our simulations, the stimulation current ranges from 120 pA to 220 pA. We consider 120 pA to be a “baseline” stimulation or background response of the neurons given that (1) there is little to no firing of either PKC*δ* or SOM neurons below this frequency (**Fig 1**) and (2) there are only minor differences in electrophysiology experiments between either PKC*δ* or SOM neurons comparing control and injured cells at this current level. It is possible to simulate the model at lower current values (<120 pA), but injury does not occur in these scenarios due to the fact that neurons do not accrue damage at current less than 120 pA.

Lastly, the global variable *Cum*_*S*_ tracks the cumulative number of time steps during which stimulation is greater than or equal to 120 pA. During initialization, this variable is set to 0, and is increased by 1 during times steps in which *S*_*i*_≥120.

### 2.4 Process overview and scheduling

The following processes occur in the order listed during each simulation of the ABM.

1. Input data is provided by user.
2. Initialization of model:
  - Create 1640 agents representing neurons in the CeA.
  - Assign attributes to all neurons.
  - Create network of directed links within each hemisphere, if network is turned on.
  - Create vector specifying stimulation history.
3. During each time step:
  - Update the cumulative stimulation variable, *Cum*_*S*_.
  - Update the damage level of each PKC*δ* and SOM neuron.
  - SOM spontaneous neurons with maximum damage (*d* = 100) are converted to regular spiking as needed based on distribution from wet lab experiments (**Fig 2**).
  - Update the firing rate of each PKC*δ* and SOM neuron using probability distributions based on neuron type, firing frequency (based on **Fig 1**), damage level, and the current stimulation value.
  - Use network to send inhibitory signals between neurons. The firing rate of each inhibited neuron is set to zero.
  - Update all system-level observations.

### 2.5 Design concepts

#### Adaptation

Individual neurons adapt to a sensitized state over time and during external stimulations measuring 120 pA or higher. This is achieved in the ABM through use of a damage accumulation model in which neurons accrue damage at individual rates and only during time steps in which *S*_*i*_≥120. As a neuron’s damage increases, the neuron transitions towards a sensitized state and its firing rate is adjusted accordingly. A neuron is considered fully sensitized when its damage variable has reached maximum value (*d* = 100).

The firing frequency of some SOM neurons changes with injury. Experimental results show the quantity of RS SOM neurons increases from 27% pre-injury to 48% post-injury, while the quantity of spontaneous SOM neurons decreases from 55% pre-injury to 34% post-injury (**Fig 2C and 2D**). We reasoned that this change in distribution may be through the actual conversion of spontaneous neurons to RS neurons. Thus, in the ABM, individual spontaneous SOM neurons that have accumulated maximum damage (*d* = 100) are converted to RS one at a time until the quantity of regular spiking SOM neurons has reached 48%.

#### Interaction

Interaction occurs in the model when a neuron is inhibited by one or more other neurons. If the sum of the inhibitory signals transmitted to a neuron during a time step exceeds 15 Hz, the neuron is inhibited (i.e., *Fr* = 0) during that time step. We chose 15 Hz as a threshold so that it is possible for a single neuron to inhibit another neuron (as reported in Hunt et al[17]). A higher threshold (e.g. 30 Hz) would be too high for any individual neuron to inhibit another neuron. A 15 Hz threshold also allows for multiple neurons with low firing rates to inhibit a target cell through their cumulative input.

#### Stochasticity

During initialization, values of damage parameters *t*_*S*_ and *t*_*L*_ are randomly determined for each neuron using a uniform probability distribution with ranges displayed in **Table 1**. Neural connectivity is achieved by creating a network of directed links between neurons using a stochastic algorithm (see Methods Section 2.7.1). During each time step, a neuron’s firing rate is stochastically updated using a weighted sum of values selected from truncated normal distributions (see Methods Section 2.7.2). Due to the stochastic nature of the model, each simulation is repeated 100 times to determine the mean, standard deviation, and confidence bounds for each output.

### 2.6 Initialization

During initialization, all 1640 agents are created. Half of all agents are assigned to the left hemisphere of the CeA; the other half are assigned to the right hemisphere. The 1600 agents representing individual neurons are assigned a protein expression type (PKC*δ* or SOM) and a firing frequency (RS, LF, or spontaneous). In our simulations, the quantities of PKC*δ* and SOM neurons within each hemisphere varied; however the proportion of RS, LF, and spontaneous neurons remained consistent across all simulations at initialization. PKC*δ* neurons are assigned firing frequencies so that 25% are LF, 48% are RS, and 27% are spontaneous consistent with our wet-lab observations (**Fig 2A**). Similarly, SOM neurons are assigned firing frequencies so that 18% are LF, 27% are RS, and 55% are spontaneous at initialization (**Fig 2C**). Additionally, individual neurons are assigned values for damage variables *t*_*L*_ and *t*_*S*_ within the ranges specified in **Table 1**. Each neuron’s damage level (*d*), number of inputs (*Num*–*In*–*Links*), and number of outputs (*Num*–*Out*–*Links*) are set to zero and all neurons are assumed to be uninhibited (*Inhibited* = *No*) during initialization. Additionally, the remaining 40 agents are each labeled as “Other.”

Once all agents have been created, a network of directed links connecting neurons to one another is established using the algorithm specified in Methods Section 2.7.1. Each directed link connects exactly two agents within the same hemisphere. Links are created during initialization only.

Lastly, during initialization, the input file specifying the stimulation history is read and the global variable *S* is created. *S* is a vector in which the *i*^th^ value indicates the stimulation current at time step *i*.

### 2.7 Submodels

#### 2.7.1 Creation of network

The stochastic algorithm described below is used to create a network of directed links between PKC*δ*, SOM, and other neurons within each hemisphere for the purpose of sending inhibitory signals. All network links are created during initialization using the probabilities displayed in **Table 3** and do not change during simulation.

**Table 3:** Probabilities associated with the creation of neural connections (e.g., directed links) transmitting signals from a PKC*δ* or SOM neuron to another neuron in the model. These probabilities were estimated from previously published data[17].

Transmitting neuron type	Receiving neuron type	Probability of Connection
PKC*δ*	PKC*δ*	0.20
PKC*δ*	SOM	0.10
PKC*δ*	Other	0.70
SOM	PKC*δ*	0.15
SOM	SOM	0.55
SOM	Other	0.30

The following algorithm describes the creation of the neural network within the left hemisphere; the same processes are repeated in the right hemisphere. The algorithm begins by randomly selecting individual neurons located in the left hemisphere until a PKC*δ* or SOM neuron with *Num-Out-Link < Max*_*out*_ is found. This neuron is identified as the transmitting neuron in a directed link. The type of agent (PKC*δ*, SOM, or other) on the receiving end of the link is randomly determined using the probabilities in **Table 3**. If the type of neuron on the receiving end of the link is either PKC*δ* or SOM, the algorithm randomly selects neurons of this type within the left hemisphere until a neuron with *Num-In-Link < Max*_*in*_ is identified. A directed link is then created from the transmitting neuron to the receiving neuron and connectivity variables (*Num-Out-Link*, *Num-In-Link*) for these two agents are updated. If the type of neuron on the receiving end of the link is “other”, a link is created from the transmitting neuron to any one of the 20 agents representing other neurons in the hemisphere and the connectivity variable (*Num-Out-Link*) of the transmitting neuron is updated. The algorithm continues to create links until all individual neurons of the same type (PKC*δ* or SOM) have achieved the maximum number of outgoing links (*Max*_*out*_) or the maximum number of incoming links (*Max*_*out*_).

#### 2.7.2 Update of damage variable for individual neurons

A damage accumulation model is used to track a neuron’s progress towards sensitization caused by noxious stimulation. During initialization, each neuron’s damage level (*d*) is set equal to 0, indicating the neuron has not accumulated any damage and is unsensitized. This is the equivalent of a naïve control animal prior to injury. A neuron accrues damage only when the cumulative amount of time under stimulation exceeds the neuron’s latency period (*Cum*_*S*_>*t*_*L*_) and the current level of stimulation is greater than or equal to 120 pA. Damage stops accumulating when it reaches its maximum value (*d* = 100), indicating the neuron is sensitized. For each individual neuron, damage at time step *i* is updated as

$$d_{i}=\left\{ \begin{array}{l} \min(d_{i-1}+\frac{100}{t_{s}},100)\;if\;CUM_{S}>t_{L}\;and\;S_{i}\geq 120\\ \;d_{i-1}\;if\;CUM_{S}\leq t_{L}\;or\;S_{i}<120 \end{array}\right.$$ (1)

where *d*_*i*_ is the value of the damage variable at time step *i*, *t*_*S*_ is the length of the neuron’s sensitization period, and *t*_*L*_ is the length of the neuron’s latency period.

#### 2.7.3 Update of firing rates for individual neurons

During each time step, the firing rates of all late firing and regular spiking neurons are stochastically updated using the equation

$${Fr}_{i}=\frac{100-d_{i}}{100}\cdot X+\frac{d_{i}}{100}\cdot Y$$ (2)

where *d*_*i*_ is the neuron’s damage level at time step *i*, and *X* and *Y* are random variables representing the firing rates of the neuron in an unsensitized state and a sensitized state, respectively. Both *X* and *Y* have truncated normal distributions defined by a mean, standard deviation, minimum value, and maximum value. Parameters depend on the neuron’s type (PKC*δ* or SOM) and firing frequency (LF or RS) as well as the stimulation level (*S_i_*). All parameter values were estimated using the data collected in the laboratory experiments outlined above (Methods Section 1.1.1) and are summarized in **S1 Table**. Eq (2) is linear combination of *X* and *Y* such that when the neuron has no damage (*d* = 0), the firing rate of the unsensitized neuron is updated using the *X* variable only. When damage reaches its maximum value (*d* = 100), the firing rate of the sensitized neuron is updated using the *Y* variable only.

Spontaneous neurons fire at a constant rate of 2.838 Hz (PKCδ) and 4.887 Hz (SOM) throughout each simulation (see Adke et al[26] and Methods Section 1.1.1 above).

#### 2.7.3 Application of network to inhibit neurons

After the firing rates of all PKC*δ* and SOM neurons are updated, the neural network is used to transmit inhibitory signals between neurons in the ABM. The strength of an inhibitory signal transmitted through a directed link is equal the firing rate of the neuron on the transmitting end. All PKC*δ* and SOM neurons are evaluated one at a time and in a random order. For each neuron, if the total strength of all incoming signals is greater than or equal to 15 Hz, the neuron is inhibited (i.e., firing rate set to zero). If the total strength is less than 15 Hz, the neuron’s firing rate does not change.

#### 2.7.4 Pain calculation

At the end of each time step, a system-level measure of pain (*P*_*i*_) is calculated as

$$P_{i}=\sum_{\begin{array}{c} Type=PKC\\ Freq=LF\;or\;RS \end{array}}\frac{d_{i}}{100}\cdot Fr_{i}-\sum_{\begin{array}{c} Type=SOM\\ Freq=LF\;or\;RS \end{array}}Fr_{i}$$ (3)

where *d*_*i*_ is a neuron’s damage and *Fr*_*i*_ is a neuron’s firing rate during time step *i*. The first summation in Eq (3) represents the weighted sum of firing rates over all PKC*δ* neurons that are either LF or RS. Each firing rate is weighted by the damage level of the corresponding neuron. As such, PKC*δ* neurons do not contribute pain when damage is zero (i.e. pre-injury), but gradually contribute to pain as sensitization occurs. When all PKC*δ* neurons have become sensitized (i.e., *d*_*i*_ = 100), each LF and RS PKC*δ* neuron contributes its firing rate to the pain calculation. The second summation in Eq (3) represents the sum of firing rates over all SOM neurons that are either late firing or regular spiking. It is assumed that SOM neurons contribute to the pain calculation at all time steps regardless of damage. Due to their anti-nociceptive properties, SOM neurons are assumed to have a negative impact on the value of pain.

### 2.8 Implementation

The model was coded in NetLogo (Version 6.0)[40]. This software has a unique programming language and customizable interface that is designed specifically for ABM development and implementation. We designed a GUI for our ABM that allows a user to easily modify parameters values, network settings, and the stimulation history. The Netlogo code and input files for simulating the ABM can be found in an Open Science Framework public repository (https://osf.io/nw5kx/, doi: 10.0.68.197/OSF.IO/NW5KX). Step-by-step instructions for downloading and simulating the model are provided in the *Supporting Information* (**S1 Text**). For the results presented here, we used BehaviorSpace within NetLogo to automate batches of 100 replicate simulations for each scenario. All graphical and statistical analyses of model output were conducted in R[41].

### 2.9 Statistics for model output

#### 2.9.1 Distributions of PKCδ and SOM firing rates

Distributions of firing rates aggregated by neuron type (PKC*δ* or SOM) resulting from model simulations were compared using a two-sided, unpaired Mann-Whitney U-Test with P<0.05 considered statistically significant. Comparisons were made between corresponding pre-injury and post-injury distributions and between corresponding left hemisphere and right hemisphere distributions. Additionally, comparisons were made between corresponding distributions resulting from model simulations with different proportions of PKC*δ* and SOM neurons.

#### 2.9.2 Standardized mean effect analysis

Standardized mean effect size analysis was completed to directly compare predicted outcomes from the three models and our wet-lab experiments. For our wet-lab experiments, we used data from published behavioral experiments designed to look at the impact of PKC*δ* and SOM inhibition with inhibitory DREADDs on mechanical sensitivity in sham (“control”) or cuff-injured mice (Methods Section 1.4 above). We replicated these wet-lab experiments using our ABM by silencing the appropriate neurons (PKC*δ* or SOM) from the model and recording pain output at times *t* = 10 (before injury) and *t* = 240 (after injury) from 5 replicate simulations. We chose to use 5 replicate model simulations to match the average *n* (sample size) from all wet-lab comparisons (mean = 4.75 ± 1.5 SD). In all simulations, we assumed a constant 120 pA current (see **Table 4** for results of these simulations; see **S3 Table** for *in vivo* data). The y-axis is different in our wet-lab experiments (mechanical sensitivity in grams) compared to the model output (arbitrary “pain” units). To normalize these axes, we calculated standardized mean effect sizes. Standardized mean effect sizes were calculated using a Hedges’ *g* value and 95% confidence intervals allowing for comparison between model outputs with constant number of replicates (*n* = 5) and wet-lab data with a variable number of samples per group (see **S4 Table** for Hedges’ g data). The Hedges’ g value was calculated as

Hedges'g=Cohen'sd×(1−34(n1+n2−2)−1), (4)

where

Cohen'sd=x1¯−x2¯(n1−1)SD12+(n2−1)SD22n1+n2−2. (5)

In the above equations, $\bar{x_{i}}$ is the mean, *n*_*i*_ is the sample size, and *SD*_*i*_ is the sample size for each group (*i* = 1, 2).

**Table 4:** Pain output from wet lab experiment (Wilson et al) and three models. Cells are shaded to indicate relative change of that individual cell from the “Intact” value found with intact PKC*δ* and SOM signaling (i.e. no inhibition). Dark green is an analgesic effect compared to intact condition and light green is a small analgesic effect compared to intact. Dark orange is a hyperalgesic effect compared to intact. No shading represents a lack of significant change from the intact value. Intact values from Wilson et al[16] are reported from two separate experiments labeled as “PKC*δ* exp” and “SOM exp”.

	Wilson et al mean (SD) units = grams*	30:70 Model mean (SD) units = arbitrary	50:50 Model mean (SD) units = arbitrary	60:40 Model mean (SD) units = arbitrary

**Uninjured**				
Intact PKC*δ* and SOM	0.81 (0.22)–*PKCδ exp* 0.41 (0.05)–*SOM exp*	-2758.38 (49.91)	-2032.10 (100.92)	-1789.40 (107.83)
PKC*δ* inhibited	0.77 (0.05)	-2904.73 (55.39)	-2110.80 (63.73)	-1868.24 (34.03)
SOM inhibited	0.03 (0.01)	0.0 (0.0)**	0.0 (0.0)**	0.0 (0.0)**

**Injured**				
Intact PKC*δ* and SOM	0.04 (0.03)–*PKCδ exp* 0.04 (0.01)–*SOM exp*	-1309.55 (114.22)	670.24 (154.31)	1295.61 (184.10)
PKC*δ* inhibited	0.66 (0.11)	-3248.85 (151.07)	-2437.36 (126.22)	-2114.26 (58.15)
SOM inhibited	0.04 (0.01)	2030.44 (74.97)	3047.86 (51.42)	3461.17 (93.37)

*Data from Wilson et al are such that high values = lower “pain” and lower values = higher “pain.” Data from computational models are such that high values = high pain and low values = lower pain.

**Mean and SD for the SOM inhibited uninjured data are zero. For calculation of Hedges’ *g* we utilized the SD of the comparison group whenever SOM inhibited uninjured data was utilized.

## Results

Our results explore model predictions of pain for a range of parameter values and scenarios. First, we simulated a default scenario in which the PKC*δ* and SOM neurons occur in equal proportions in the CeA. We use this scenario to demonstrate the model’s ability to produce changes in pain during and after injury that are consistent with our expectations. Additional simulations of the model under this scenario were performed to explore the impact of select model parameters on system-level output, such as pain and total number of inhibited neurons.

Second, we simulated the model using the proportions of PKC*δ* and SOM neurons that were obtained from wet-lab experiments. We found differences in the relative proportion of PKC*δ* and SOM across these studies and used the model to explore the impact of these differences on predicted values of pain. To validate our model predictions, we calculated the standardized mean difference between values of pain outputted from the model and those observed in wet-lab experiments.

### Simulation of pain using ABM with equal proportions of PKC*δ* and SOM neurons

Previously published data as well as new wet lab data were used to estimate values of parameters in the ABM. In our published data set[16], we demonstrated changes in excitability of labeled PKC*δ* and SOM neurons in the right CeA under control and chronic cuff injury conditions (**Fig 1**). Neurons were classified as either regular spiking (RS) or late firing (LF). Broadly speaking, the excitability of PKC*δ* neurons increased with injury and the excitability of SOM neurons decreased with injury. These data are in line with our behavioral observations when we manipulated PKC*δ* or SOM neurons using chemogenetic vectors. In those data, we found a pro-nociceptive output from PKC*δ* neurons and an anti-nociceptive output from SOM neurons. For the purposes of the ABM, we assumed that pain output was dependent on a balance between pro-nociceptive PKC*δ* and anti-nociceptive SOM neurons. When necessary, we extrapolated our physiology data to a maximum of 220 pA using a linear model (**Fig 1** triangles). We also measured the proportions of RS, LF, and spontaneous neurons in slices from control and injured mice (**Fig 2**). While PKC*δ* neuron proportions did not drastically change after injury (**Fig 2A and 2B**), we did observe an apparent change in proportion of SOM neurons in injured mice (**Fig 2C and 2D**). We found more RS and fewer spontaneous SOM neurons in injured mice compared to the control mice.

**Fig 3:**
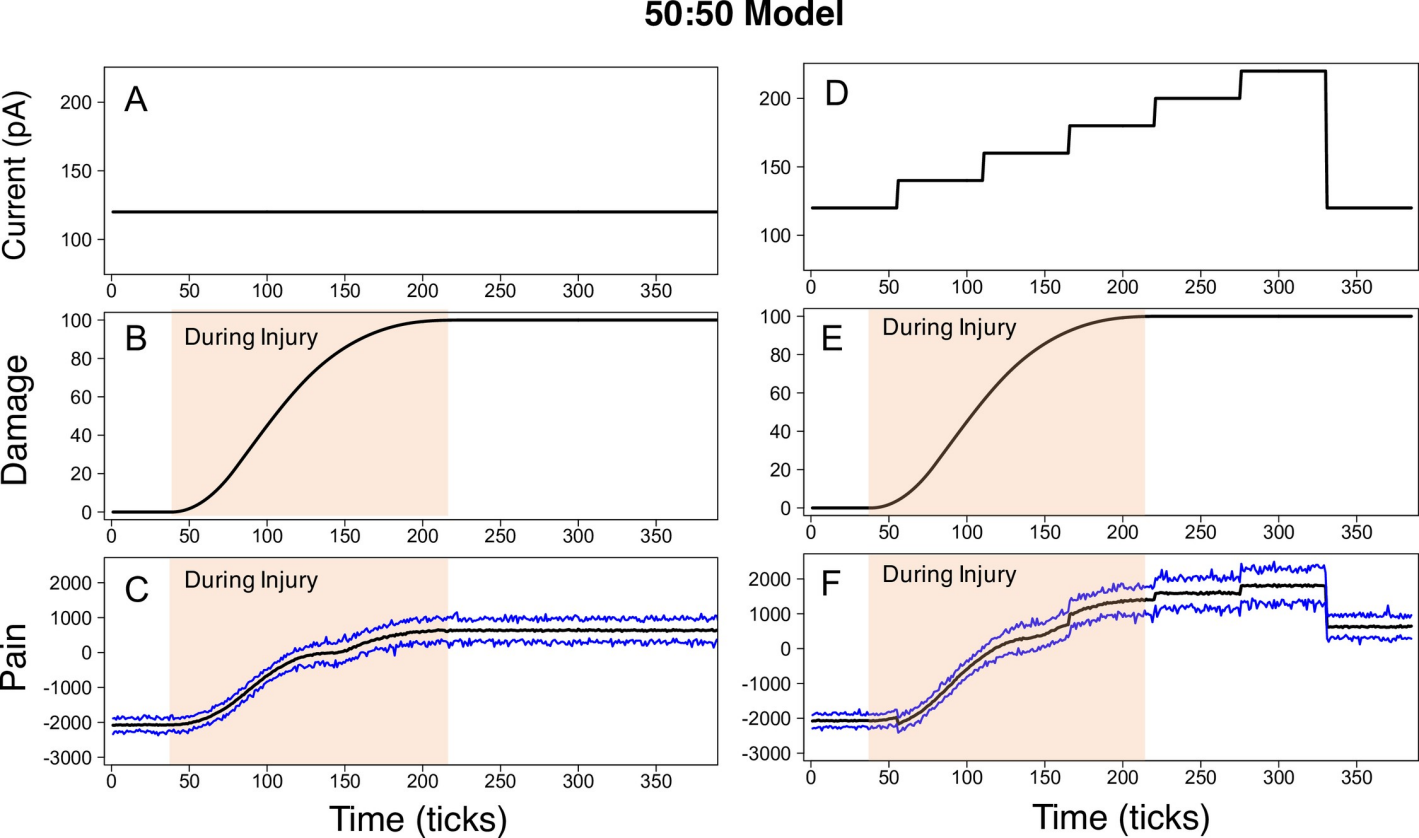
50:50 model output during constant and ramping current scenarios. The 50:50 model was simulated using constant 120 pA current (**A**) and ramping current (**D**). In the model, PKC*δ* and SOM neurons accumulate damage during injury. Average damage values from all PKC*δ* and SOM neurons during the constant current (**B**) and ramping current (**E**) scenarios are displayed. In both scenarios, pain increases during injury and remains elevated post-injury. The average (black line), minimum (bottom blue line), and maximum (top blue line) model pain output across 100 replicate simulations is displayed for the constant current (**C**) and ramping current (**F**) scenarios.

Initial simulations of the ABM were performed to determine if the model could replicate these increases in electrophysiological output after injury and in response to changes in external stimulation. In these simulations, the model was initialized with an equal number of PKC*δ* and SOM neurons in each of the left and right CeAs (referred to as the “50:50” model). Additionally, during the creation of the connectivity network, each neuron was allowed a maximum of three inputs (*Max*_*in*_ = 3) and three outputs (*Max*_*out*_ = 3). Emergent values of “damage” and “pain” were analyzed for two different stimulation scenarios. In the first scenario, the external stimulation is constant 120 pA for the duration of the simulation (**Fig 3A**). In the second scenario, the external stimulation starts at 120 pA and gradually ramps up to 220 pA before returning to 120 pA at the end of the simulation (**Fig 3D**). 120 pA can be conceptually thought of in an animal as spontaneous “pain.” We would expect little spontaneous pain in control mice but more in injured mice. In contrast, 220 pA (peak current value) would yield the equivalent of the type of “pain” experienced by an animal if it were given a strong noxious pinch. Both the control and injured mice would experience an increase in “pain” to pinch but the injured animal’s response would be classified as hyperalgesic. Due to the stochasticity of the ABM, each scenario was repeated 100 times. The average damage across all neurons and replicate simulations was calculated for the constant 120 pA scenario and the ramping current scenario. Injury occurs during damage accumulation (shaded regions in **Fig 3B** and **3E**).

In both scenarios, pain output (difference between cumulative PKC*δ* and SOM firing rates on the left and right) significantly increased above baseline measures during injury and remained elevated post-injury. **Fig 3** shows the average, maximum and minimum pain values observed over the 100 replicate simulations for the constant 120 pA scenario (**Fig 3C**) and the ramping current scenario (**Fig 3F**). Average pain during baseline conditions (i.e., before injury) is approximately -2000 in both scenarios, indicating a dominant presence of antinociceptive output from SOM neurons and an absence of pain. During injury, average neural damage increased steadily until it reached maximum value (*d* = 100). At the same time, pain increased to a positive value and remained elevated after injury. In interpreting the model’s pain output, our focus is on the change in pain values over time rather than the absolute values of pain on the y-axis. The positive changes in pain values during injury are attributed to the increasing pro-nociceptive output from the PKC*δ* neurons and the elevated pain values that remain after injury suggest this pro-nociceptive activity outweighs any anti-nociceptive output from the SOM neurons when injury is present. Previous studies have shown that endogenous pain pathways tonically inhibit pain signals at baseline in well-known areas in the brainstem[42] as well as in the amygdala[9,16]. The net negative values at baseline in our model are consistent with this baseline tonic inhibition, primarily driven by SOM neurons in our model.

In the scenario with ramping current, pain output fluctuated in response to changes in the external stimulation. With each 20 pA increase in current, pain also increased (**Fig 3F**). At maximum current (220 pA), pain ranged from 1000 to 2500. When current was dropped back down to 120 pA at the end of the simulation, pain decreased (compared to the 220 pA level) but still remained significantly higher than baseline measures. Overall, one can think of the model’s predictions of pain before injury as the spontaneous pain experienced by an animal at baseline (i.e. no pain). During injury, damage accumulates and neurons become sensitized, causing an increase in pain. Increases in stimulation (e.g. >120 pA) lead to increases in pain. As described above, this can be thought of as the evoked pain caused by “pinching” the paw of an animal. Finally, after injury has occurred and the stimulation has been reduced to 120 pA, the elevated pain output is considered spontaneous non-evoked pain experienced after injury.

### Model simulations demonstrate the importance of neuronal circuits in pain output

Using the 50:50 model, we explored the impact of connectivity between neurons in the CeA. In designing the connectivity network, we utilized published data to estimate the number of connections between neurons. Based on data showing an average of 3.5 primary dendrites in CeC neurons[29], we considered four different networks of primary connections (e.g. primary dendrites or primary axonal branches): no connectivity (0:0 network), exactly 1 input and 1 output per neuron (1:1 network), at most 3 inputs and 3 outputs per neuron (3:3 network), and at most 5 inputs and 5 outputs per neuron (5:5 network). The maximum number of inputs (*Max*_*in*_) and maximum number of outputs (*Max*_*out*_) per neuron did not differ between PKC*δ* and SOM neurons. To estimate the probability of PKC*δ*-to-PKC*δ*, PKC*δ*-to-SOM, SOM-to-SOM, and SOM-to-PKC*δ* connections, we utilized another published data set[17]. In those experiments, the authors used a combination of transgenic expression markers and immunohistochemistry to determine the frequency of PKC*δ*-to-PKC*δ*, PKC*δ*-to-SOM, PKC*δ*-to-other, SOM-to-SOM, SOM-to-PKC*δ* and SOM-to-other connections. To extract these data from the paper, we calculated the percentage of all connections between classified neurons in the manuscript (n = 20), of which 55% were SOM-to-SOM, 20% were PKC*δ*-to-PKC*δ*, 10% were PKC*δ*-to-SOM, and 15% SOM-to-PKC*δ*. These values correspond to the probability of a PKC*δ*-to-PKC*δ*, PKC*δ*-to-SOM, SOM-to-SOM, and SOM-to-PKC*δ* connection in the model (**Table 3**). All other connections in the model are assumed to be either PKC*δ*-to-Other or SOM-to-Other where “Other” indicates a neuron that is neither PKC*δ* nor SOM and does not contribute to pain output.

**Fig 4:**
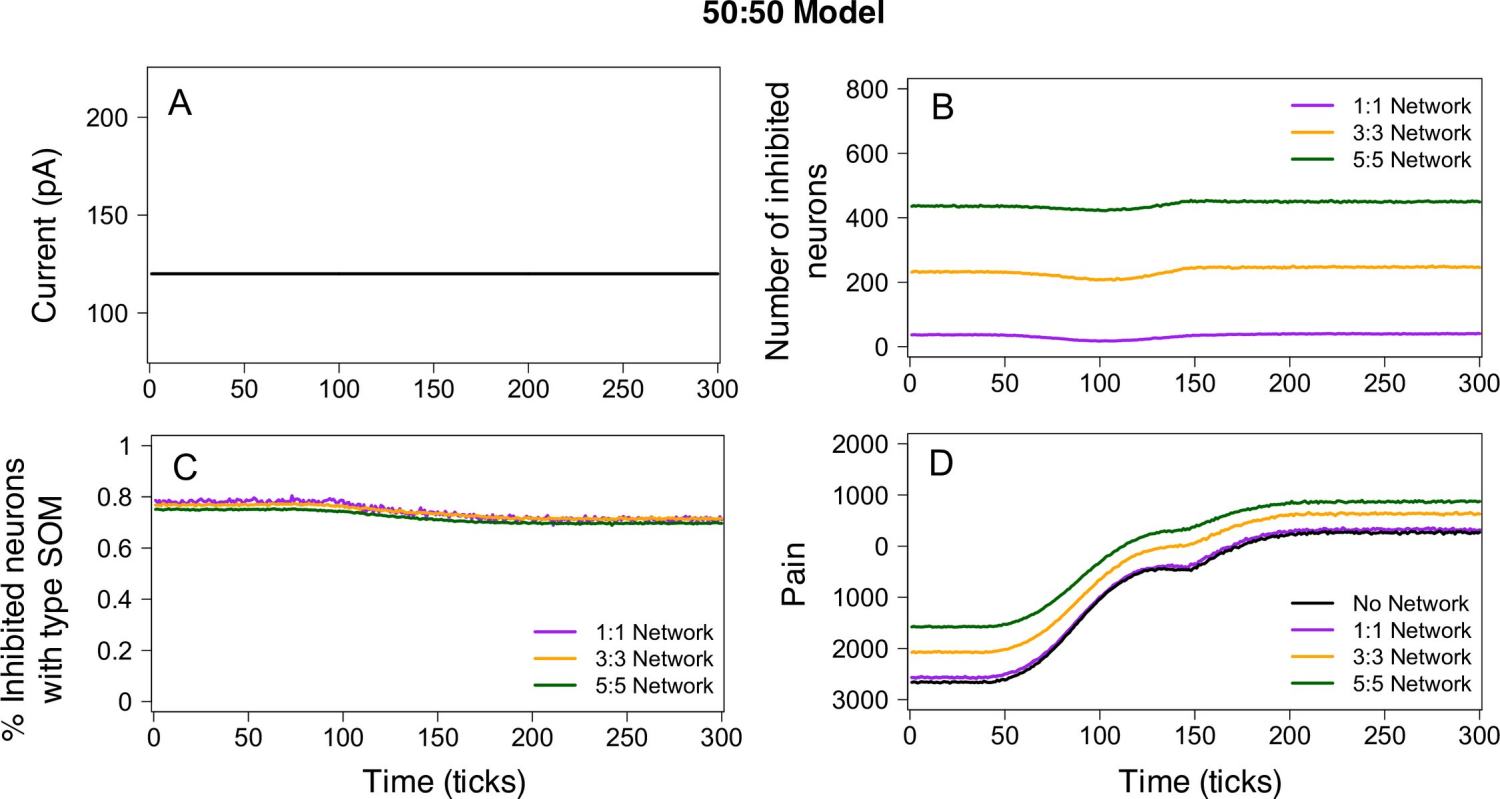
50:50 model output for different network parameters. Simulations of the 50:50 model were performed for four different connectivity networks: No network, 1:1 network, 3:3 network, and 5:5 network. In all simulations, we assumed a constant 120 pA current (**A**). The total number of inhibited neurons (**B**) and pain output (**D**) varied across the different network structures. However, the percentage of inhibited neurons that are SOM was similar for the 1:1, 3:3, and 5:5 networks (**C**). In simulations with no network, there are no inhibited neurons and the pain output closely resembles pain output from the 1:1 model.

We explored the impact of the network parameters *Max*_*in*_ and *Max*_*out*_ on model output by simulating the constant 120 pA stimulation scenario for four different network configurations (0:0, 1:1 3:3, and 5:5) (**Fig 4**). The total number of links in each network increased as values of *Max*_*in*_: *Max*_*out*_ increased. The 1:1 network yielded an average of 1600 links, the 3:3 network yielded an average of 4764 links, and the 5:5 network yielded an average of 7879 links in the network. Similarly, larger values of *Max*_*in*_: *Max*_*out*_ resulted in a larger number of inhibited neurons at any time point (**Fig 4B**). Across all networks with links (1:1, 3:3, and 5:5), approximately 60–80% of inhibited neurons were SOM (**Fig 4B**), with the remaining inhibited neurons being PKC*δ*. In the 0:0 network, no links exist and therefore no neurons are inhibited at any time. Average pain values were similar for model simulations using the 0:0 (no network) and 1:1 network. However, due to the large number of inhibited “anti-nociceptive” SOM neurons, higher levels of pain were observed in model simulations using the 3:3 and 5:5 networks (**Fig 4D**). Given the presence of actual cell-to-cell networks in real life, the baseline pain outputs of these last two models are likely more accurate and represent the important contribution of the network on what a baseline pain state actually is *in vivo*.

### Model output is most impacted by the proportions of PKC*δ* and SOM neurons

We next wanted to understand the impact of select parameters on the model’s prediction of pain before, during, and after injury. To achieve this goal, we performed a local sensitivity analysis. For the sensitivity analysis, we initialized the 50:50 model with a 3:3 network configuration and assumed a constant stimulation of 120 pA throughout each simulation. Parameters included in the sensitivity analysis and their baseline values are: PKC*δ*-to-PKC*δ* connectivity probability (0.20), PKC*δ*-to-SOM connectivity probability (0.10), SOM-to-SOM connectivity probability (0.55), SOM-to-PKC*δ* connectivity probability (0.15), percentage of SOM neurons labeled as RS (27%), percentage of PKC*δ* neurons labeled as RS (48%), and the percentage of SOM neurons in each hemisphere (50%). For each parameter, an upper endpoint was identified as *R*^+^ = *R*+0.05 and a lower endpoint was identified as *R*^−^ = *R*−0.05, where *R* represents the baseline value of the parameter. To generate sensitivity values, the model was simulated 100 times for each parameter when evaluated at the lower endpoint, baseline value, and upper endpoint. In each simulation, values of pain (*P*) were outputted at times *t* = 10 (before injury), *t* = 105 (during injury), *t* = 240 (after injury). At each of these three timepoints, the sensitivities of pain to each parameter were calculated as $S^{+}=\frac{\left(P^{+}-P\right)}{\left(R^{+}-R\right)}$ and $S^{-}=\frac{\left(P^{-}-P\right)}{\left(R-R^{-}\right)}$ where *P*^−^, *P*, *P*^+^ are the average values of pain when the parameter is valued at *R*^−^, *R*, *R*^+^, respectively. Here, *S*^+^ represents the sensitivity of model outputted values of pain to an increase in the parameter and *S*^−^ represents the sensitivity of pain to a decrease in the parameter.

**Fig 5:**
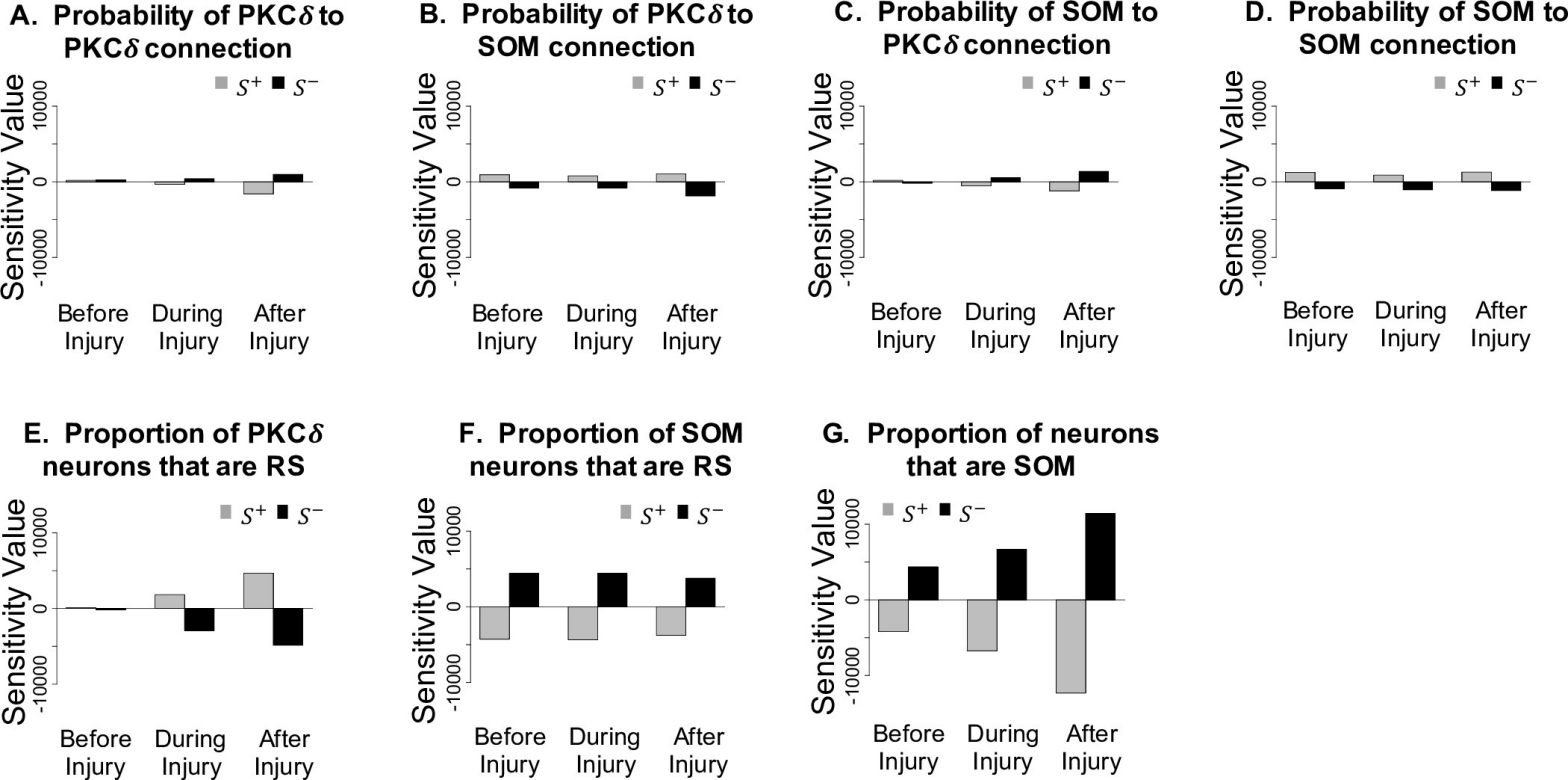
Sensitivity analysis of 50:50 model. A sensitivity analysis was conducted on the 50:50 model with constant 120 pA current to determine the sensitivity of pain output to select model parameters before injury (t = 10), during injury (t = 105), and after injury (t = 240). Values of *S*^+^ and *S*^−^ represent the sensitivity of pain to small increases and decreases, respectively, in each parameter. Relatively small values of *S*^+^ and *S*^−^ are associated with the connectivity parameters (**A-D**). The larger sensitivity values associated with the parameters determining the proportion of PKC*δ* neurons that are regular spiking (**E**), the proportion of SOM neurons that are regular spiking (**F**), and the proportion of neurons that are SOM (**G**) indicate pain is most sensitive to perturbation in these parameters.

**Fig 5** displays the sensitivity values (*S*^+^, *S*^−^) before, during, and after injury for each of the seven parameters. The y-axis of all plots in **Fig 5** is the same for comparison across parameters. Sensitivity values are largest for the parameter controlling the relative quantity of SOM and PKC*δ* neurons (**Fig 5G**). The gray bars indicate that a small increase to the percentage of neurons that are SOM (and therefore a decrease to the percentage of neurons that are PKC*δ*) will result in a substantial decrease in pain, especially during and after injury. Similarly, the black bars indicate that a small decrease to this parameter will result in a substantial increase to pain, especially during and after injury.

Sensitivity values were moderately high for the parameters controlling the proportion of PKC*δ* and SOM neurons that are RS (**Fig 5E and 5F**). Surprisingly, the network connectivity parameters (**Fig 5A and 5D**) had the smallest sensitivity values, indicating that small changes to these parameters have relatively less impact on pain.

### Model simulations using alternative proportions of PKC*δ* and SOM neurons highlight a current controversy in the literature

Motivated by the results of the sensitivity analysis, we next wanted to probe the effects of differential expression of PKC*δ* and SOM neurons in both the right and left amygdala. The difference in the relative quantity of PKC*δ* and SOM cells has been hypothesized as an important factor in whether the amygdala sends an increased pain signal or decreased signal; however, there is controversy in the literature on the magnitude of differences in these cell types in the amygdala[17,18]. We simulated the model for three different assumptions regarding the percentage of PKC*δ* and SOM neurons in the CeA.

**Fig 6:**
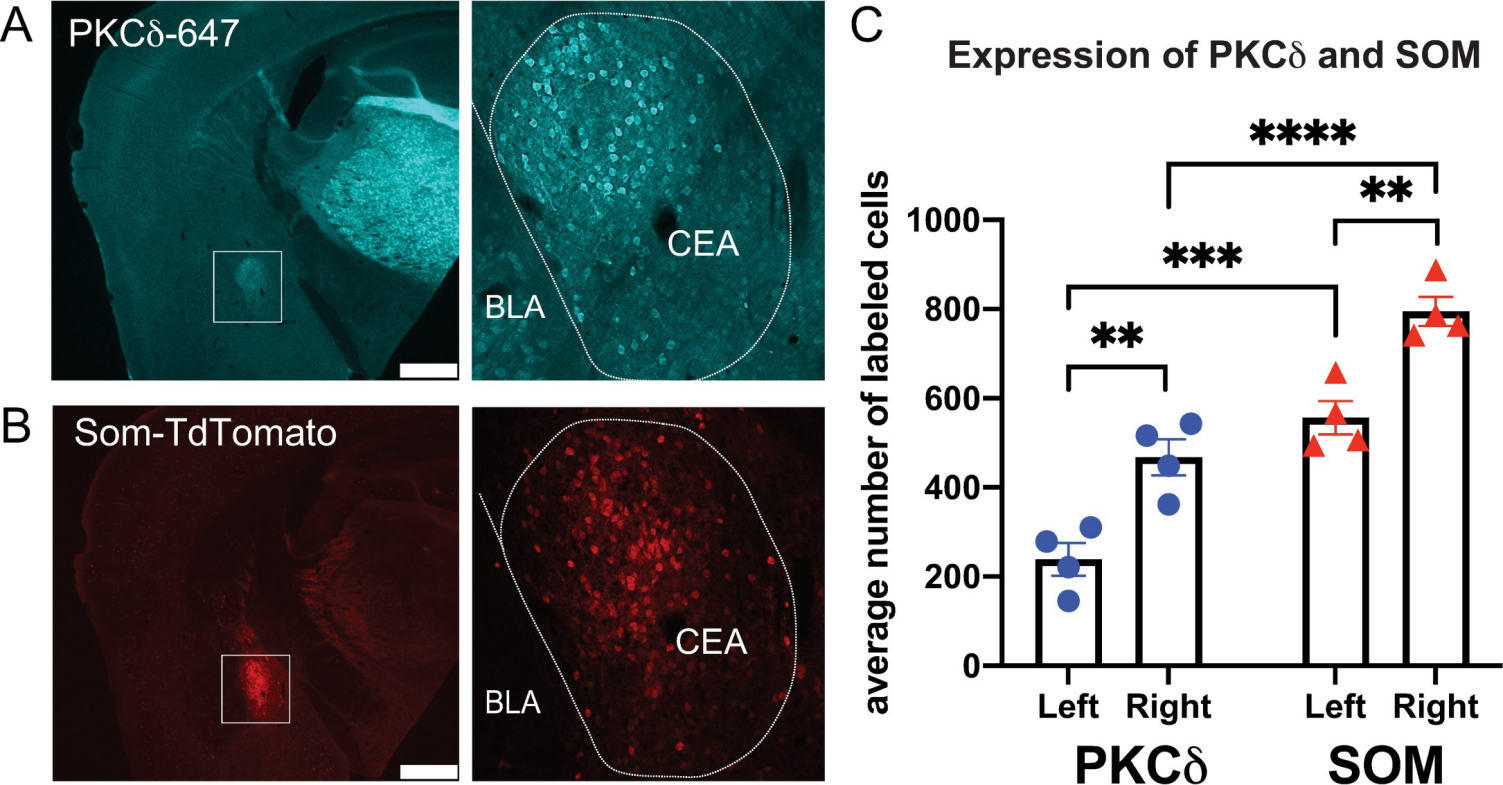
Expression of PKC*δ* and SOM in the left and right CeA. Utilizing Ai9 reporter mice for SOM and immunohistochemistry for PKC*δ*, the average number of (**A**) PKC*δ* and (**B**) SOM neurons were counted in the left or right CeA from -0.58 to -1.94mm Bregma. Images show 2x of left hemisphere along with 10x zoomed image of the CeA and basolateral amygdala (BLA). Scale bar = 500μm. (**C**) There are significantly more PKC*δ* neurons on the right CeA compared to the left. There are significantly more SOM neurons on the right CeA compared to the left. There are significantly more SOM vs PKC*δ* neurons on the left and right CeA. Two-Way RM ANOVA with Sidak multiple comparisons ** P<0.01, ** P<0.001, *** P<0.0001.

As described above, the 50:50 results were obtained using an equal number of PKC*δ* and SOM neurons in each the left and right hemisphere. Additionally, the ABM was simulated with initial distributions of PKC*δ* and SOM neurons matching those observed in two separate laboratory experiments. First, the model was initialized with 56% PKC*δ* and 44% SOM in each the left and right hemispheres, as was reported in Hunt et al 2017[17]. These model conditions are referred to as the “60:40” model. Second, the model was initialized with 30% PKC*δ* and 70% SOM in the left hemisphere and 37% PKC*δ* and 63% SOM in the right hemisphere. This model is referred to as the “30:70” model. The percentages in the 30:70 model were based on our new analysis of left and right PKC*δ* and SOM expression data (**Fig 6**). Comparing left and right CeA PKC*δ* and SOM, we found significant main effects of side of brain (2-way RM ANOVA P = 0.007) and cell type (P = 0.0001). Multiple comparisons showed a statistically significantly higher number of PKC*δ* neurons (**Fig 6A**) on the right compared to the left CeA (**Fig 6C**; P<0.01). We found a statistically significantly higher number of SOM neurons (**Fig 6B**) on the left compared to the right CeA (**Fig 6C**; P<0.01). Finally, there were significantly more SOM neurons compared to PKC*δ* on the left and the right (**Fig 6C**; P<0.001).

**Fig 7:**
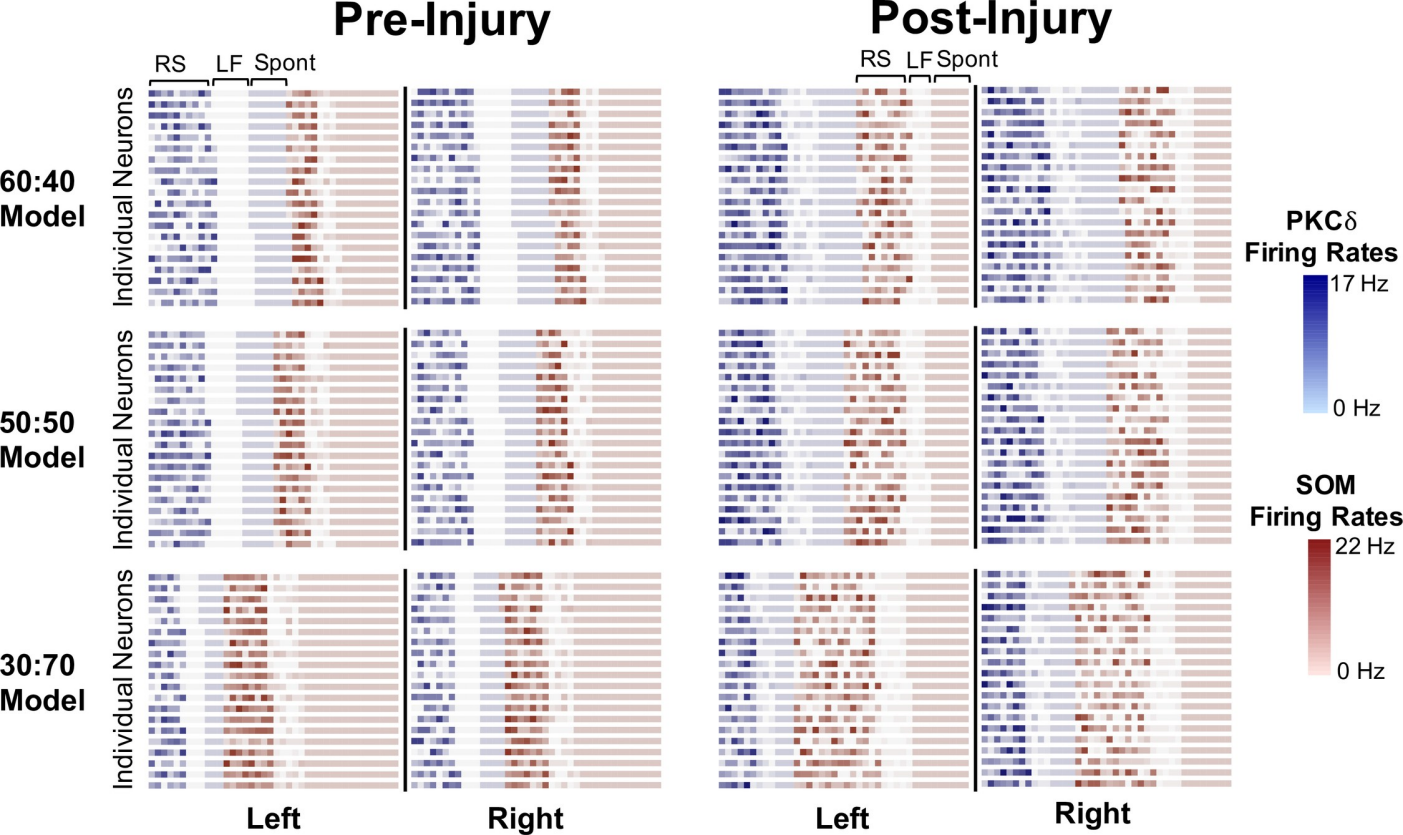
Individual neuron firing rates before and after injury. For each of our three models, the firing rates of all 1600 neurons during a simulation with constant 120 pA were outputted before injury (t = 10) and after injury (t = 240). Darker hues correspond to higher firing rates in PKC*δ* neurons (blue) and SOM neurons (red). Regular spiking (RS), late firing (LF), and spontaneous (Spont) SOM and PKC*δ* neurons are grouped together in each plot. Across all three models, the firing rates of PKC*δ* RS and LF neurons increase with injury and the firing rates of SOM RS and LF neurons decrease with injury (corresponding to lab data in **Fig 1**). However, injury also results in an increase in the quantity of SOM RS neurons and a decrease in the quantity of SOM spontaneous neurons (corresponding to lab data in **Fig 2**). Data on the left and right are the same for the 60:40 and 50:50 models, but distinct for the 30:70 model (corresponding to lab data in **Fig 6**).

We repeated 100 replicate model simulations for each of these three sets of initial conditions (50:50, 60:40, and 30:70). In all simulations, we used the 3:3 network parameters described above and assumed a constant 120 pA external simulation. First, we inspected differences in individual-level neural activity before and after injury across the three models (50:50, 60:40, 30:70). For each model, we outputted the firing rate (obtained prior to inhibition by the network) and firing frequency (RS, LF, or Spontaneous) of all 1600 PKC*δ* and SOM neurons at time *t* = 10 (before injury) and time *t* = 240 (after injury) from one of the 100 replicate simulations. We then plotted each neuron in the left CeA as a square on a 40x20 grid, and likewise for the right CeA, with color corresponding to the neuron’s type (blue = PKC*δ*, red = SOM) and hue determined by the neuron’s firing rate (**Fig 7**). For the purpose of this visualization, neurons were grouped by firing frequency within each hemisphere.

Several distinguishing characteristics of the three models are visible in **Fig 7.** First and foremost, the difference in quantity of PKC*δ* and SOM neurons across the three models is evident by the different proportion of blue and red squares, respectively. Second, the top two rows corresponding to the 60:40 model and 50:50 model show no difference in the distribution of PKC*δ* and SOM neurons when comparing the left and right hemispheres. However, the bottom row shows an asymmetric distribution of PKC*δ* and SOM neurons that is unique to the 30:70 Model. In all three models, injury causes a decrease in the quantity of SOM spontaneous neurons and an increase in the quantity of SOM RS. This is most visible in 30:70 model due to the large number of SOM neurons present.

**Fig 8:**
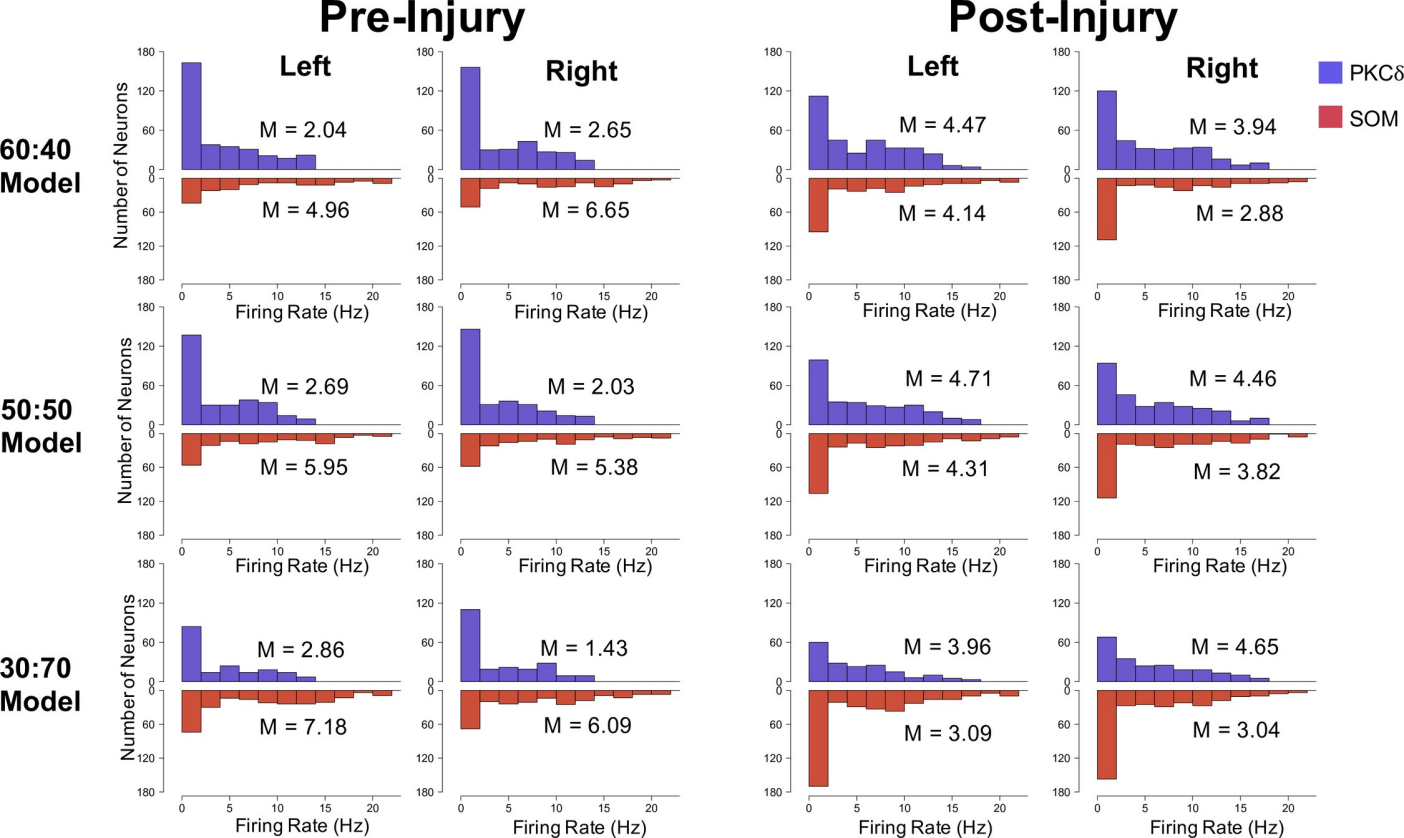
Aggregated PKC*δ* and SOM firing rates before and after injury. Histograms show the distributions of firing rates for all PKC*δ* RS and LF neurons (blue) and all SOM RS and LF neurons (red) before injury (*t* = 10) and after injury (*t* = 240) during constant 120 pA stimulation. The median firing rate (M) is provided for each distribution. In all three models (50:50, 60:40, 30:70), the post-injury firing rates of PKC*δ* neurons are significantly higher than their corresponding pre-injury firing rates (P < 0.05) and the post-injury firing rates of SOM neurons are significantly less than their corresponding pre-injury firing rates (P < 0.05) with the exception of left side SOM neurons in the 50:50 model (P = 0.053). There are no statistically significant differences in the corresponding distributions of firing rates between hemispheres (Left vs. Right) or across models (e.g., 50:50 vs. 60:40).

Second, we aggregated the firing rates of all RS and LF neurons in **Fig 7** by type (PKC*δ* or SOM) and compared corresponding distributions within and across the three models. **Fig 8** shows the distributions of firing rates for all PKC*δ* RS and LF neurons (blue) and all SOM RS and LF neurons (red) in each hemisphere before and after injury. Statistical comparisons were made between corresponding pre-injury and post-injury distributions, between corresponding left and right distributions, and between distributions resulting from different models (e.g. 60:40 vs. 30:70). In almost all situations, we found a significant difference in the firing rates of corresponding pre-injury and post-injury distributions with one exception (50:50 model for left CeA SOM) which showed a strong trend (P = 0.053). As expected, there was a decrease in the firing rates of SOM RS and LF neurons after injury and an increase in the firing rates of PKC*δ* RS and LF neurons after injury. None of the other comparisons resulted in statistically significant differences between corresponding distributions. Thus, while the quantity of PKC*δ* and SOM neurons differed between the models, the distributions of their firing rates did not. See **S2 Table** in the Supporting Information for the U-values and P-values of all comparisons.

**Fig 9:**
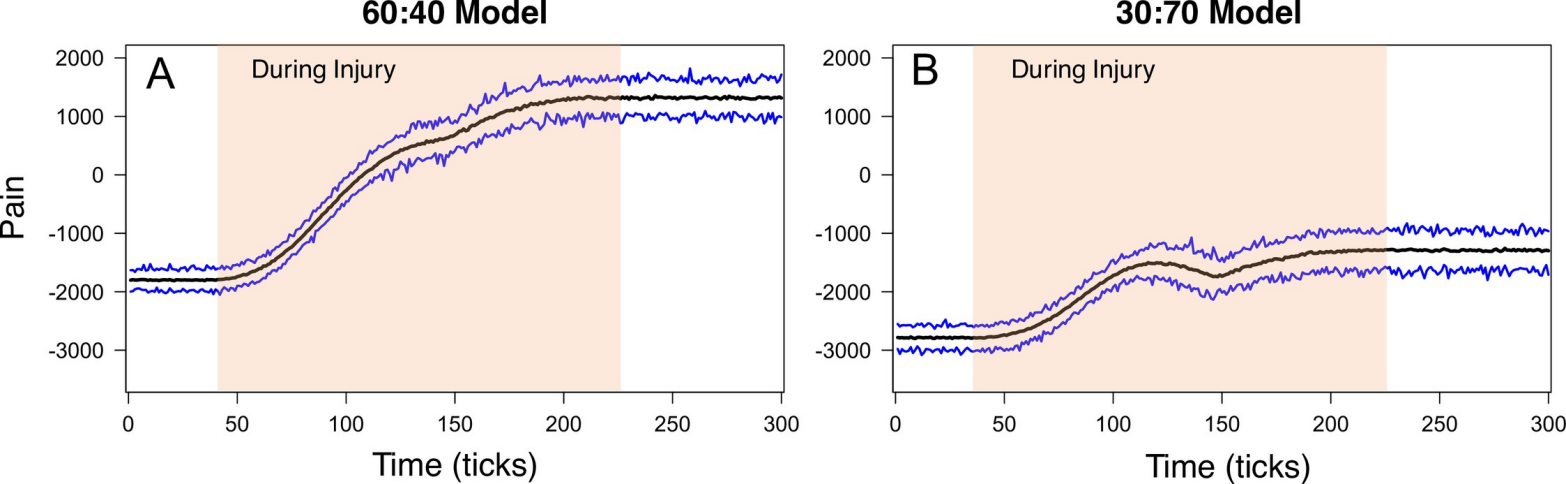
60:40 and 30:70 model output. The 60(PKC*δ*):40(SOM) model (**A**) and 30:70 model (**B**) were each simulated 100 times using constant 120 pA current and the average, minimum, and maximum pain output was plotted. In both models, pain increases during injury and remains elevated post-injury. However, in the 30:70 model a brief decrease in pain is observed around time *t* = 125 as spontaneous SOM neurons are converted to regular spiking.

Third, we compared system-level measures of pain across the three different models. Pain output differed dramatically between the model versions (**Fig 9**). Pain values from the 60:40 model (**Fig 9A**) were slightly higher than those in the 50:50 model (**Fig 3C**) due to a small increase in PKC*δ* neurons (pro-nociceptive output) and small decrease in SOM neurons (anti-nociceptive output). The 30:70 model did not produce the same pattern of pain development observed in the other models. In the 30:70 model, average pain output is approximately -2800 during baseline (pre-injury) conditions and increases to approximately -1200 during injury (**Fig 9B**). Overall, the 30:70 model yielded the smallest change in pain during injury compared to the other models. Negative pain values throughout the simulation of the 30:70 model are attributed to the large quantity of SOM neurons in both the left and right hemispheres. Pain actually decreases at one point during injury when 21% of SOM neurons (~224 neurons) are converted from spontaneous to regular spiking. The stark difference in pain observed across these two models (**Fig 9**) corroborate the results of the sensitivity analysis (**Fig 5**) showing that changes in the relative proportions of PKC*δ* and SOM neurons have a large impact on pain. Moreover, the fact that changes in the relative proportions of PKC*δ* and SOM neurons in CeA led to the greatest changes in pain suggests that this parameter could be used as a metric to understand chronic pain plasticity and/or treatment.

### Standardized effect analyses demonstrate good concordance between model predicted effect sizes and observed effect sizes in mice

After building our three models, we sought to determine whether predicted outcomes from the models would match our wet-lab data using the cuff neuropathic pain model in male mice[16]. We utilized Hedges’ g standardized mean effect size analysis to compare these data sets.

**Fig 10:**
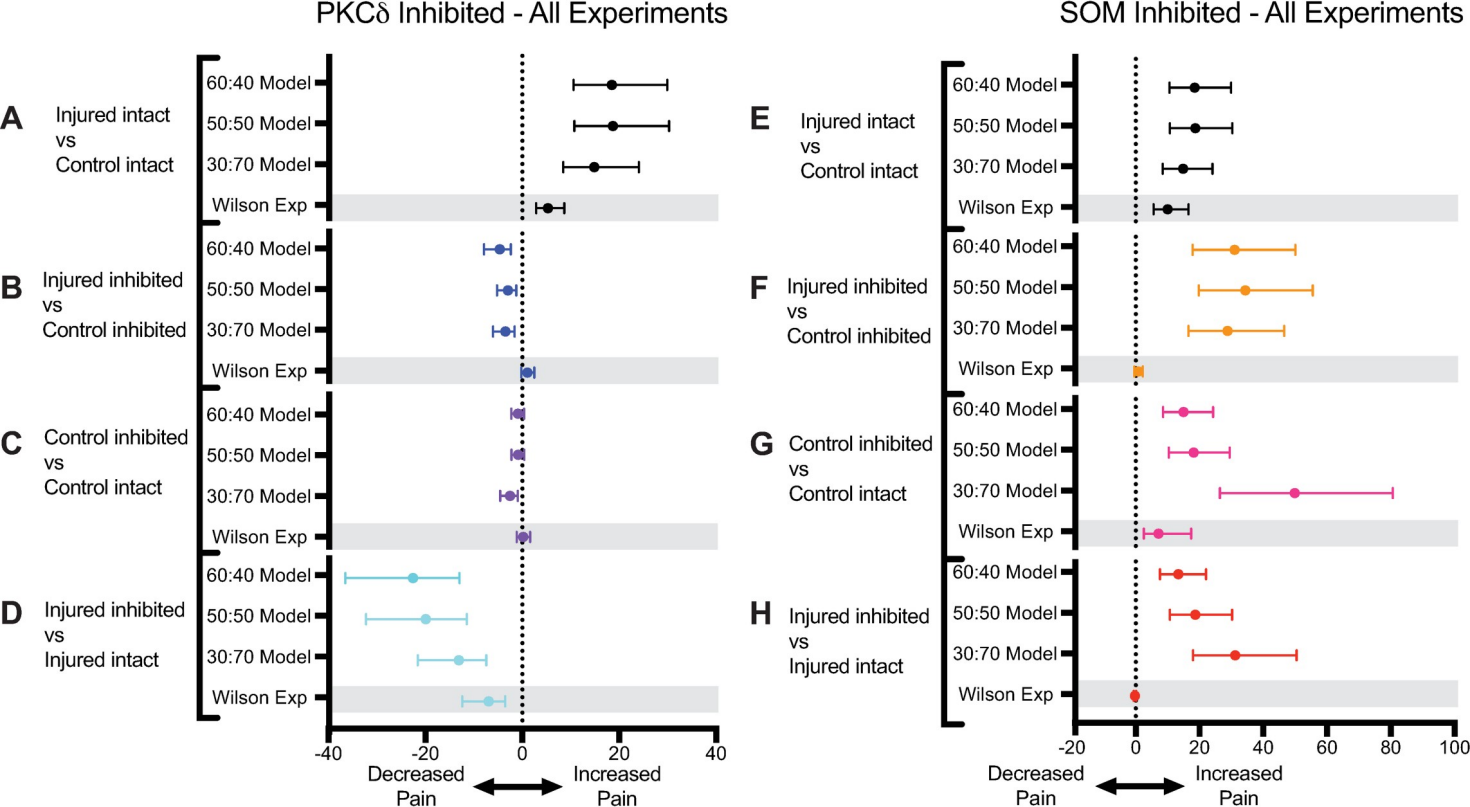
Standardized mean effect differences between wet-lab experiment and model simulations. Hedges’ *g* standardized mean effect differences were calculated between different groups from our wet-lab experiments and three models (60:40, 50:50, and 30:70) which differed based on the PKC*δ*:SOM ratio. Forest plots show Hedges’ *g* +/- 95% confidence intervals. Positive Hedges’ *g* is indicative of an increase in pain-like effect for the indicated comparison and a negative Hedges’ *g* is indicative of a decrease in pain-like effects. (**A, E**) Hedges’ *g* calculations for intact experiment when neither PKC*δ* nor SOM is manipulated. Black data points show the standardized difference between control groups and injured groups with strong congruency across the wet-lab experiment and all three models showing that injury/damage accumulation leads to an increase in “pain” compared to control condition. (**B-D**) Data showing the impact of PKC*δ* inhibition. (**B**) Blue data points show the impact of PKC*δ* inhibition in both the control and injured groups. (**C**) Purple data points show the impact of PKC*δ* inhibition in the control group only. (**D**) Light blue data points show the impact of PKC*δ* inhibition in the injured group only. (**F-H**) Data showing the impact of SOM inhibition. (**F**) Orange data points show the impact of SOM inhibition in both the control and injured groups. (**G**) Pink data points show the impact of SOM inhibition in the control group only. (**H**) Red data points show the impact of SOM inhibition in the injured group only. Wet lab data are from published work[16].

For the inhibition of each PKC*δ* and SOM, we completed four different standardized mean difference scores using Forest Plots with Hedges’ *g* and 95% confidence intervals (CIs) plotted (**Fig 10**). In these calculations, any mean difference greater than zero is indicative of increased “pain.” First, we compared the standardized mean difference of injured and uninjured pain output values. As expected, we found positive values for our wet-lab data and all three models (**Fig 10A and 10E**). The magnitude of the pain effect in the models is generally close or within the 95% CIs of the wet-lab data although the Hedges’ g for the model data were slightly higher. The magnitude of the model pain output increases slightly as the proportion of PKC*δ* cells increases and SOM decreases. So, the highest magnitude is with the 60(PKC*δ*):40(SOM) model followed by the 50:50 model, and the 30:70 model has the lowest magnitude. Next, we compared Hedges’ *g* values between control and injured animals when either PKC*δ* or SOM was inhibited.

For PKC*δ*, we found two areas of congruency and one area of difference between the models and the wet-lab data (**Fig 10B, 10C and 10D**). First, a similar pattern was seen when evaluating the Hedges’ *g* for inhibited control groups versus uninhibited (intact) control groups (**Fig 10C**; purple data points). Second, we evaluated the Hedges’ *g* comparing injured groups with or without PKC*δ* inhibition (**Fig 10D**; light blue data points). Here, we found an “analgesic” effect of PKC*δ* inhibition for the wet-lab experiment and all three models with overlapping CI’s. Finally, we investigated the impact of inhibiting PKC*δ* in control and injured conditions with the hypothesis that these groups would likely be similar under this inhibitory condition. For the wet-lab data, there was no meaningful difference between the control and injured mice when PKC*δ* was inhibited (**Fig 10B**; “Wilson et al” data points). Hedges’ *g* effect sizes for the three models, in contrast, demonstrated a small mean difference that was less than zero (i.e. a decrease in pain).

Next, we replicated these analyses with inhibition of SOM (**Fig 10F, 10G and 10H**). When inhibiting SOM in injured and control groups, we found no significant effect size for the wet-lab data but substantial increases in effect size for all three models (**Fig 10F**; orange data points). In the control condition comparing SOM inhibition to intact SOM, we see good alignment in the Hedges’ *g* between the wet-lab experiment and the three models (**Fig 10G**; pink data points). Finally, when evaluating SOM inhibition in the injured mice (**Fig 10H**; red data points), we see a similar pattern to what is seen for inhibition in the control vs inhibition in the injured condition with all three models in agreement (overlapping CI’s) but in contrast to the null effect observed in the wet-lab data (**Fig 10F**).

## Discussion

In this paper, we present an agent-based model (ABM) of pain-related neurons in the left and right hemispheres with a focus on the CeA. Each neuron in the model is described by a type (PKC*δ* or SOM), firing frequency (LF, RS, or spontaneous), and location (left or right CeA). Neural firing rates are stochastically updated at each time step and inhibitory signals are transmitted between neurons via the connectivity network. During periods of noxious stimulation, neurons accrue damage which, over time, leads to sensitization and changes in the neurons’ firing rates. A main purpose of the ABM is to determine how these changes at the neuron-level impact system-level responses. The primary system-level measure in our study is “pain,” which we defined as the difference between cumulative pro-nociceptive neural output and anti-nociceptive neural output across the left and right hemispheres.

The ABM presented here is an expansion of our preliminary model[10] and offers new insight into the interplay of SOM and PKC*δ* neurons that could not have been afforded by the previous version. The current model offers greater heterogeneity at the neuron-level by including physiological and histological properties of neurons as well as a connectivity network allowing neurons to transmit inhibitory signals to one another. While the preliminary model only allowed for painful stimulation to be ‘on’ or ‘off’, the current model allows for stimulation to any take value in a defined range (e.g. 120 pA to 220 pA in our simulations). Thus, the current ABM is able to output values of pain that evolve over time *and* in response to varying levels of stimulation. As stimulation (i.e. current) increases, the average individual firing rates of SOM and PKC*δ* neurons increase (**Fig 1**). Although PKC*δ* and SOM neurons have opposite roles in pain modulation, increases to their firing rates do not result in a zero net change to pain. As seen in **Fig 3F**, increases in current result in corresponding increases to pain (i.e. evoked pain) despite there being an equal number of SOM and PKC*δ* neurons. This phenomena is attributed to the presence of the network in the ABM and the fact that the majority of inhibited neurons are SOM (**Fig 4C**).

When possible, the parameter values in our ABM were estimated from previously published studies, and when this was not possible, we performed our own wet-lab experiments to gather the necessary data. One parameter in particular emerged as highly influential in determining pain output. This parameter is the relative proportion of PKC*δ* neurons to SOM neurons, and, in our model this parameter is allowed to vary between the left and right hemispheres. To minimize the bias of this parameter in our model output, our initial simulations assumed a 50:50 ratio (50% PKC*δ* and 50% SOM in both hemispheres). Under this scenario, our model showed promising results by predicting an increase in pain during injury and elevated pain levels post-injury. Subsequently, we used the 50:50 model to explore the impact of the connectivity network on pain output. These results showed that the 1:1 network and 0:0 network (no-network) yield similar results. However, as the complexity and size of the network increased (e.g., 3:3, 5:5 network), we observed more inhibited neurons and higher pain values compared to output from the 1:1 and 0:0 networks. This increase in pain is attributed to the fact that a majority of the inhibited neurons are SOM (anti-nociceptive). When SOM is inhibited, the remaining pro-nociceptive PKC*δ* agents increase “pain” output. These results suggest the connectivity network plays an important role in modulating pain and the inclusion of this feature in our model is important. Our model included only primary inputs/outputs and did not account for secondary and tertiary branches in neurons. We find that while the number of primary dendrites is not different between PKC*δ* and SOM cells, the number of tertiary branches increases in SOM CeA neurons[26]. This will be an important factor to consider in future versions of the network.

Another variable to consider in future versions of the model is the spatial relationship between connections. As seen in the modeling of the lateral nucleus of amygdala output in Pavlovian fear memories, distance between different neurons is a major factor in overall model output[43]. Within the CeA, functional connections between and within PKC*δ* and SOM neurons have been found to be ~50–100 um[17] but dendrites/axons from these neurons are on the order of 500–1000 um[15,17] suggesting the potential for longer range connections that have yet to be found. Ultimately, additional wet-lab experiments are necessary to better understand the structure and size of the CeA network between PKC*δ* and SOM neurons. This would entail a more thorough paired recording setup that specifically looks for both short-range and long-range projections with post-hoc cell filling dyes to evaluate direct versus indirect connections at baseline and following injury.

We used both published data and the results of our own wet-lab experiments to estimate the proportion of PKC*δ* and SOM neurons in the CeA. First, we found significant left-right lateralization, which has not been previously reported. Second, our data highlighted a current controversy in the literature. We found higher numbers of SOM vs PKC*δ* neurons in the CeA, which is consistent with another report[18] that found twice as many SOM neurons as PKC*δ* (side of brain undefined). In contrast, two other reports have found more PKC*δ* neurons than SOM neurons[17,39]. One possible and important distinction between approaches was Hunt et al’s[17] and Kovner et al’s[39] use of direct immunodetection for both PKC*δ* and SOM compared to Han et al’s[18] and our own analyses that used a Cre-dependent reporter line for SOM, coupled with immunodetection for PKC*δ*[18]. In our experiment, any neuron that has ever expressed SOM in development will continue to express the reporter even if that neuron no longer expresses SOM. In that context, we and others may have overestimated the quantity of SOM neurons.

A major assumption of the model as written is that all PKC*δ* are pro-nociceptive and all SOM neurons are anti-nociceptive. This assumption followed our wet-lab experiments in which the primary output from manipulation of these two populations in naïve and injured mice demonstrated such a dichotomy on pain-like behavior. However, there is evidence that some PKC*δ* neurons may be anti-nociceptive. Isoflurane gas anesthesia activates a group of CeA neurons[11]. When additionally activated with DREADDS, these neurons reduced pain-like behavior in mice. It was reported that 79% of these neurons expressed PKC*δ*. Future studies will be critical in determining the relative proportion of PKC*δ* neurons that drive pro-nociceptive versus anti-nociceptive output from the CeA. A related variable in these studies will be the physical location of the neurons within the CeA. Data from appetitive and defensive behaviors has found differences in the roles of neuronal sub-types depending on the subnuclei of the CeA[44]. Fortunately, such factors, when available, would be straightforward to incorporate into our ABM.

To evaluate the ABM we directly compared the effect size of model predictions of pain under different conditions to our previously collected data from mice[16]. First, without manipulating PKC*δ* or SOM, all three of our models fit the wet-lab nerve injury pain model output in both direction (e.g. an increase in “pain” after injury) and magnitude. Second, we inhibited the PKC*δ* or SOM neurons by silencing their firing rates. This action can easily be achieved using the “inhibitory” buttons in the NetLogo GUI (see **S1 Text and S1 Fig**). This interface is a major advantage of using the NetLogo software to develop the ABM. The inhibitory sliders are the equivalent of both classic pharmacology approaches using receptor antagonists and newer approaches using inhibitory opto- and chemo-genetics.

When we inhibited PKC*δ* neurons in our three models (50:50, 60:40, 30:70), we found areas of congruency and incongruency. In mice, PKC*δ* inhibition had no effect in control animals. When we looked at the standardized mean effects for inhibition of control groups, we found that two of the three models and the wet-lab data had CIs that straddled the zero line (i.e. a null difference; **Fig 10C**). Only the 30:70 model showed a very slight analgesic effect of PKC*δ* inhibition in the control condition.

In mice, PKC*δ* inhibition nearly completely reversed the cuff model mechanical hypersensitivity[16]. Thus, it was not surprising that the Hedges’ *g* for the wet-lab data had a confidence interval that straddled the zero Hedges’ *g* line (**Fig 10B**). In contrast to this wet-lab data, Hedges’ *g* calculation of all three models demonstrated a small “analgesic” mean difference that is attributed to a small impact of PKC*δ* inhibition in the control condition but with a consistent large impact of PKC*δ* inhibition in the injured condition for the models. The lack of an effect in control wet-lab mice with PKC*δ* inhibition may be related to the behavioral assay used in our previous publication, namely mechanical sensitivity analysis. Mechanical stimulation is not a suprathreshold nocifensive response in naïve mice. If wet-lab experiments were completed in naïve mice using a hyperalgesic stimulus such as hot plate, inhibition of PKC*δ* in control mice may very well reduce the behavioral response aligning these data with the model predictions. On the other hand, it is possible that *in vivo* behavioral assays are simply too coarse to detect small analgesic effects with typical experimental variability. Overall, the analgesic effect in the models was modest compared to the larger Hedges’ *g* seen with our comparison of PKC*δ* inhibition in injured groups versus intact (no PKC*δ* inhibition) injured groups.

In that experiment, we found large effects sizes and good concordance between the wet-lab mice and all three models. Essentially, inhibition of PKC*δ* reversed the effects of injury (damage accumulation). This is consistent with a largely pro-nociceptive role for PKC*δ* after injury. The magnitude of the effect size for the models was similar to the wet-lab experiment with two of three models having overlapping CI’s with the wet lab CI’s (**Fig 10D**).

Similar to PKC*δ*, when we inhibited SOM neurons in our three models (50:50, 60:40, 30:70), we found areas of incongruency and congruency with our wet-lab findings. We believe that the incongruent aspects again may be driven by limitations in the animal assays. First, for inhibition of control and injured SOM neurons our models predicted an increase in pain consistent with an anti-nociceptive output of SOM neurons; the Hedges’ *g* for the wet-lab data showed a zero effect size. Here, the difference between the models and the wet-lab data is likely attributed to the fact that the actual mice are at the “floor” in the mechanical assay while the model output has no floor. Injured mice are at floor in the actual behavior assay (i.e. maximum hypersensitivity) before SOM inhibition and cannot experience more “pain” with loss of analgesic SOM cells. In uninjured mice, SOM inhibition causes so much mechanical hypersensitivity that those animals are also at floor. Thus, there is a null Hedges’ *g* mean difference. These animals though are not likely at a biological maximum for pain, it is simply a disadvantage of that assay. In contrast, in our models without a floor, we see that inhibition of SOM in the injured group causes a larger hyperalgesic difference in Hedges’ *g* compared to inhibition of SOM in the control condition. A similar explanation can be applied to differences in the model prediction for SOM inhibition in the injured state versus intact (no SOM inhibition) injured groups (**Fig 10H**). That is, the wet-lab data show no difference between the intact injured mice and the SOM inhibited injured mice because the mice are at floor in the assay and cannot show any greater mechanical hypersensitivity. In contrast, our model output, with no floor, is able to show that loss of SOM causes an increase in pain like-effects in the context of on-going injury. This assay floor issue did not confound our interpretation of Hedges’ *g* analysis comparing SOM inhibition in the control condition versus intact (no SOM inhibition) in the control condition (**Fig 10G**). In all circumstances, wet-lab and models, inhibition of SOM in the control groups causes a hyperalgesic reaction compared to the intact control group and two of three models have overlapping CIs with the wet-lab CIs. The only model to not overlap with the wet-lab data was the 30:70 model which showed the highest hyperalgesic effect. Since this model has the largest number of SOM positive neurons, it is not surprising that it also shows the biggest effect of losing those neurons. All of the mean effect data are consistent with the idea that SOM provides analgesic tone such that when it is removed through inhibition, the mouse/model shows increased pain-like effects.

If we look across all of our simulations and experiments, we can make some observations regarding which model topologies fit the *in vivo* wet lab data the best. It is clear that the 3:3 and 5:5 networks are more in line with our understanding of the connectivity in the CeA than 1:1 and no networks. Using the 3:3 network, we varied the proportion of PKC*δ* and SOM neurons based on existing experimental data and our new experimental data. As described, there is good concordance within the models in **Fig 10** with two notable exceptions. In **Fig 10C**, we see that 60:40 and 50:50 perfectly match up with the wet-lab data but the 30:70 model shows an analgesic change. In a similar manner, in **Fig 10G**, we see stronger matching between the 60:40 and 50:50 models with the wet-lab data than we do for the 30:70 model. Here, the 30:70 model shows a greater increase in pain when SOM is inhibited in control conditions. Overall, these data all point to the 60:40 model (PKC*δ*>SOM) with a 3:3 network as being more accurate of the real-world scenario compared to the 30:70 model (PKC*δ*<SOM).

The ABM presented here has two distinct functions for the field moving into the future. First, the ABM serves a central framework for synthesizing data across multiple research studies and detecting emergent properties of the amygdala that might not be obvious in direct experimental testing. As researchers continue to amass data and formulate hypotheses at the neuron-level, this information can be included in the ABM framework. Subsequently, through simulation and formal analyses, the ABM can be used to evaluate the collective properties of neurons and in particular, the impact of these properties on pain. Second, the ABM can be immediately used by researchers to complete “thought” experiments prior to implementation of time-intensive and laborious wet-lab experiments. The Netlogo interface (**S1 Fig**) is straightforward and the software is free. Using a combination of the inhibition and expression sliders (e.g., proportion of PKC*δ* vs SOM), investigators can explore the interaction of PKC*δ* and SOM in the left and right amygdala (**S1 Text**). As described in the introduction, left versus right differences in amygdala function have come to represent a major consideration in the interpretation of wet-lab experiments[8]. Comparison of the different network models can also be used to evaluate the impact of synaptic plasticity that might occur in disease states.

Overall, this ABM of the amygdala represents the first comprehensive model of the amygdala in the context of pain using modern cell-type specific electrophysiological and expression data. Future iterations of the model will incorporate additional cell markers (e.g., corticotropin-releasing factor (CRF), calcitonin gene-related peptide (CGRP) receptor, dynorphin/kappa opioid receptor (KOR), pituitary adenylate cyclase-activating peptide receptor (PAC1), etc.) and finer gradations of proportions of cells driving anti-nociceptive and pro-nociceptive output from the amygdala in the context of injury and pain.

## Supporting information

S1 Fig Graphical user interface for ABM model built using NetLogo. (**A)** Panel illustrates a sample screenshot of the NetLogo user interface at the end of a model simulation. Features of the interface include monitors, plots, buttons, toggle switches, a pull-down menu, and sliders to allow a user to control the parameters of a simulation and observe the output produced by the model. A 40x41 grid of patches and agents (white arrows) represent the CeA and its neurons. Within this grid, the boundary between the hemispheres is a grey vertical line and neurons are assigned a color based on their role in the CeA as follows: SOM RS neurons are blue, SOM LF neurons are light blue, PKCδ RS neurons are red, PKCδ LF neurons are light red, all spontaneous neurons are black, “other” neurons are grey, and inhibited neurons are yellow. Panel also contains the buttons used to initialize and then start a simulation. Drop-down menu allows user to select a current stimulation history file. (**B**) Zoom in of left CeA sliders to control the proportions of neurons (PKC*δ* vs SOM) and firing types. (**C**) Zoom in depicts a close-up of a toggle switches to control the inclusion of a neural network and sliders to control the firing rates of spontaneous neurons, inhibition threshold input signal, and maximum inputs and outputs a neuron can have. Panel also contains the buttons used to silence certain groups of neurons, which can be pressed any time after the Initialize button is pressed. (**D**) During a simulation, the total pain output is graphed in real-time. Representative graph shows pain output status near the end of a simulation. Panel portrays a close-up of a plot outputted by the model, which tracks the changes in pain value over time. (**E**) Other outputs in real-time include left vs right CeA total pain output (not shown) and left (shown) and right (not shown) PKC*δ* vs SOM cumulative firing rates over time. The purple curve represents the PKCδ RS and LF neurons. The blue curve represents the SOM RS and LF neurons.
(TIFF)

S1 Table Parameters defining the probability distributions for random variables X (unsensitized firing rate) and Y (sensitized firing rate) in Eq (2). Both X and Y have truncated normal distributions with mean *μ*, standard deviation *σ*, minimum value *min*, and maximum value *max*.
(DOCX)

S2 Table Results of statistical tests comparing distributions of PKC*δ* and SOM firing rates. A two-tailed, unpaired Mann-Whitney test was used to compare corresponding distributions of firing rates from PKC*δ* and SOM neurons in the left and right hemispheres before and after injury (as seen in **Fig 8**). The table below provides the results (U-value and P-value) of the Mann-Whitney tests. *P<0.05.
(DOCX)

S3 Table Raw data from Wilson et al used for mean effect size calculations. Low values are indicative of mechanical hypersensitivity. Mean and SD of each group is given at baseline (before injection) and 1 hour after injection of the DREADD activator Clozapine N-oxide (CNO) or control saline (Sal).
(DOCX)

S4 Table Hedges’ *g* values calculated for *in vivo* studies and three models. (DOCX)

S1 Text Step-by-step directions for simulating the agent-based model in NetLogo. (DOCX)
