# Supplementary figures and images for: Agent-based modeling of the central amygdala and pain using cell-type specific physiological parameters
Source: PLoS Comput Biol. 2021 Jun 8;17(6):e1009097. doi: 10.1371/journal.pcbi.1009097 (PMC8213159; doi:10.1371/journal.pcbi.1009097)

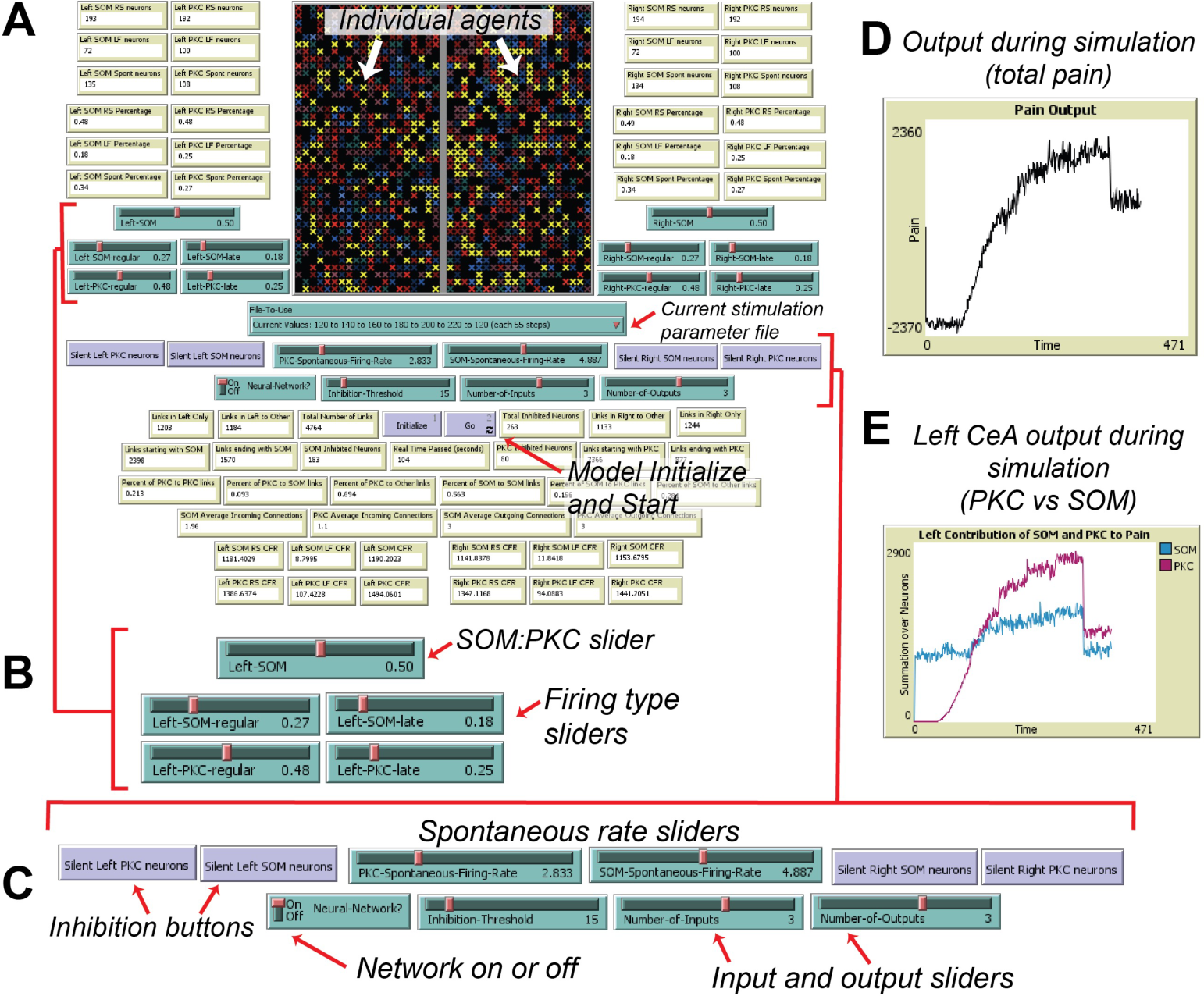

Supplement: S1 Fig — (A) Panel illustrates a sample screenshot of the NetLogo user interface at the end of a model simulation. Features of the interface include monitors, plots, buttons, toggle switches, a pull-down menu, and sliders to allow a user to control the parameters of a simulation and observe the output produced by the model. A 40x41 grid of patches and agents (white arrows) represent the CeA and its neurons. Within this grid, the boundary between the hemispheres is a grey vertical line and neurons are assigned a color based on their role in the CeA as follows: SOM RS neurons are blue, SOM LF neurons are light blue, PKCδ RS neurons are red, PKCδ LF neurons are light red, all spontaneous neurons are black, “other” neurons are grey, and inhibited neurons are yellow. Panel also contains the buttons used to initialize and then start a simulation. Drop-down menu allows user to select a current stimulation history file. (B) Zoom in of left CeA sliders to control the proportions of neurons (PKCδ vs SOM) and firing types. (C) Zoom in depicts a close-up of a toggle switches to control the inclusion of a neural network and sliders to control the firing rates of spontaneous neurons, inhibition threshold input signal, and maximum inputs and outputs a neuron can have. Panel also contains the buttons used to silence certain groups of neurons, which can be pressed any time after the Initialize button is pressed. (D) During a simulation, the total pain output is graphed in real-time. Representative graph shows pain output status near the end of a simulation. Panel portrays a close-up of a plot outputted by the model, which tracks the changes in pain value over time. (E) Other outputs in real-time include left vs right CeA total pain output (not shown) and left (shown) and right (not shown) PKCδ vs SOM cumulative firing rates over time. The purple curve represents the PKCδ RS and LF neurons. The blue curve represents the SOM RS and LF neurons. (TIFF) [file pcbi.1009097.s001.tiff]
