# Supplementary material for: Agent-based modeling of the central amygdala and pain using cell-type specific physiological parameters
Source: PLoS Comput Biol. 2021 Jun 8;17(6):e1009097. doi: 10.1371/journal.pcbi.1009097 (PMC8213159; doi:10.1371/journal.pcbi.1009097)
Supplement: S1 Table — Both X and Y have truncated normal distributions with mean μ, standard deviation σ, minimum value min, and maximum value max. (DOCX) [file pcbi.1009097.s002.docx]

**S1 Table: Parameters defining the probability distributions for random variables X (unsensitized firing rate) and Y (sensitized firing rate) in equation (2).** Both X and Y have truncated normal distributions with mean $\mu,$standard deviation $\sigma$, minimum value $min$, and maximum value $max$.

| **Type** | **Firing Freq.** | **Stimulation (pA)** | **Random Variable** $\boldsymbol{X}$ | | | | **Random Variable** $\boldsymbol{Y}$ | | | |
| --- | --- | --- | --- | --- | --- | --- | --- | --- | --- | --- |
|  |  |  | $\boldsymbol{\mu}$ | $\boldsymbol{\sigma}$ | $\boldsymbol{min}$ | $\boldsymbol{max}$ | $\boldsymbol{\mu}$ | $\boldsymbol{\sigma}$ | $\boldsymbol{min}$ | $\boldsymbol{max}$ |
| PKC$\delta$ | LF | 0 – 60 | 0 | 0 | 0 | 0 | 0 | 0 | 0 | 0 |
| PKC$\delta$ | LF | 80 | 0 | 0 | 0 | 0 | 0.0833 | 0.2887 | 0 | 1 |
| PKC$\delta$ | LF | 100 | 0 | 0 | 0 | 0 | 0.25 | 0.6216 | 0 | 2 |
| PKC$\delta$ | LF | 120 | 0 | 0 | 0 | 0 | 0.9167 | 1.505 | 0 | 5 |
| PKC$\delta$ | LF | 140 | 0.1111 | 0.3234 | 0 | 1 | 2 | 2.0889 | 0 | 7 |
| PKC$\delta$ | LF | 160 | 0.3889 | 0.6978 | 0 | 2 | 3 | 2.5226 | 0 | 8 |
| PKC$\delta$ | LF | 180 | 0.8889 | 1.1827 | 0 | 3 | 4.25 | 2.958 | 1 | 10 |
| PKC$\delta$ | LF | 200 | 1.3889 | 1.4608 | 0 | 4 | 5.3333 | 2.9336 | 2 | 11 |
| PKC$\delta$ | LF | 220 | 2.1667 | 1.9778 | 0 | 6 | 6.75 | 3.1659 | 3 | 13 |
| PKC$\delta$ | RS | 0 | 0 | 0 | 0 | 0 | 0 | 0 | 0 | 0 |
| PKC$\delta$ | RS | 20 | 0.1111 | 0.4646 | 0 | 2 | 0.2778 | 0.6691 | 0 | 2 |
| PKC$\delta$ | RS | 40 | 0.5278 | 1.483 | 0 | 6 | 0.8333 | 1.6891 | 0 | 5 |
| PKC$\delta$ | RS | 60 | 1.2778 | 2.2375 | 0 | 8 | 1.8889 | 2.8052 | 0 | 7 |
| PKC$\delta$ | RS | 80 | 2.4444 | 3.1932 | 0 | 10 | 3.3333 | 3.9705 | 0 | 12 |
| PKC$\delta$ | RS | 100 | 3.9167 | 4.0169 | 0 | 13 | 5.3889 | 4.6291 | 0 | 15 |
| PKC$\delta$ | RS | 120 | 5.5833 | 4.5127 | 0 | 14 | 7.6111 | 4.6544 | 1 | 17 |
| PKC$\delta$ | RS | 140 | 7.1389 | 4.9289 | 0 | 15 | 9.6111 | 4.6417 | 2 | 19 |
| PKC$\delta$ | RS | 160 | 8.75 | 5.1513 | 1 | 16 | 10.571 | 4.6417 | 2 | 19 |
| PKC$\delta$ | RS | 180 | 10.0833 | 5.261 | 1 | 18 | 12.18 | 4.6417 | 2 | 19 |
| PKC$\delta$ | RS | 200 | 11.4444 | 5.2777 | 2 | 20 | 13.789 | 4.6417 | 2 | 19 |
| PKC$\delta$ | RS | 220 | 12.6389 | 5.3353 | 3 | 21 | 15.398 | 4.6417 | 2 | 19 |
| SOM | LF | 0 - 80 | 0 | 0 | 0 | 0 | 0 | 0 | 0 | 0 |
| SOM | LF | 100 | 0.375 | 1.0607 | 0 | 3 | 0 | 0 | 0 | 0 |
| SOM | LF | 120 | 1 | 2.4495 | 0 | 7 | 0.1429 | 0.378 | 0 | 1 |
| SOM | LF | 140 | 1.5 | 3.2071 | 0 | 9 | 0.4286 | 1.1339 | 0 | 3 |
| SOM | LF | 160 | 2.5 | 4 | 0 | 11 | 1.1429 | 2.2678 | 0 | 6 |
| SOM | LF | 180 | 3.25 | 4.7734 | 0 | 13 | 1.8571 | 2.9114 | 0 | 8 |
| SOM | LF | 200 | 4.5 | 5.6315 | 0 | 15 | 3.1429 | 3.9761 | 0 | 11 |
| SOM | LF | 220 | 5.625 | 5.7554 | 0 | 16 | 4.4286 | 4.237 | 0 | 12 |
| SOM | RS | 0 – 20 | 0 | 0 | 0 | 0 | 0 | 0 | 0 | 0 |
| SOM | RS | 40 | 0.3077 | 1.1094 | 0 | 4 | 0.45 | 1.2763 | 0 | 5 |
| SOM | RS | 60 | 2.0769 | 2.6914 | 0 | 8 | 1.75 | 3.3067 | 0 | 11 |
| SOM | RS | 80 | 4.6154 | 4.0935 | 0 | 13 | 3.45 | 4.8284 | 0 | 15 |
| SOM | RS | 100 | 8.0769 | 4.9068 | 1 | 17 | 5.15 | 6.1239 | 0 | 19 |
| SOM | RS | 120 | 11.2308 | 5.5551 | 2 | 22 | 7 | 7.0038 | 0 | 21 |
| SOM | RS | 140 | 13 | 4.6726 | 4 | 23 | 8.51 | 7.0038 | 0 | 21 |
| SOM | RS | 160 | 14.3077 | 4.9897 | 5 | 28 | 10.16 | 7.0038 | 0 | 21 |
| SOM | RS | 180 | 15.9231 | 6.1028 | 6 | 30 | 11.81 | 7.0038 | 0 | 21 |
| SOM | RS | 200 | 16.7692 | 6.2471 | 7 | 30 | 13.46 | 7.0038 | 0 | 21 |
| SOM | RS | 220 | 17.1538 | 5.97 | 8 | 31 | 15.11 | 7.0038 | 0 | 21 |
