# Supplementary material for: Agent-based modeling of the central amygdala and pain using cell-type specific physiological parameters
Source: PLoS Comput Biol. 2021 Jun 8;17(6):e1009097. doi: 10.1371/journal.pcbi.1009097 (PMC8213159; doi:10.1371/journal.pcbi.1009097)
Supplement: S2 Table — A two-tailed, unpaired Mann-Whitney test was used to compare corresponding distributions of firing rates from PKCδ and SOM neurons in the left and right hemispheres before and after injury (as seen in Fig 8). The table below provides the results (U-value and P-value) of the Mann-Whitney tests. *P<0.05. (DOCX) [file pcbi.1009097.s003.docx]

**S2 Table: Results of statistical tests comparing distributions of PKC**$\boldsymbol{\delta}$ **and SOM firing rates.** A two-tailed, unpaired Mann-Whitney test was used to compare corresponding distributions of firing rates from PKC$\delta$ and SOM neurons in the left and right hemispheres before and after injury (as seen in **Fig 8**). The table below provides the results (U-value and P-value) of the Mann-Whitney tests. *P<0.05

| **Cell Population** | **Comparison** | **U** | **P** |
| --- | --- | --- | --- |
| 50:50 Model PKC$\delta$ Left CeA | Pre-Injury vs. Post-Injury | 30694 | *< 0.0001 |
| 50:50 Model SOM Left CeA | Pre-Injury vs. Post-Injury | 26611 | 0.05352 |
| 50:50 Model PKC$\delta$ Right CeA | Pre-Injury vs. Post-Injury | 29028 | *< 0.0001 |
| 50:50 Model SOM Right CeA | Pre-Injury vs. Post-Injury | 26818 | *0.02535 |
| 60:40 Model PKC$\delta$ Left CeA | Pre-Injury vs. Post-Injury | 37756 | *< 0.0001 |
| 60:40 Model SOM Left CeA | Pre-Injury vs. Post-Injury | 21282 | *0.01084 |
| 60:40 Model PKC$\delta$ Right CeA | Pre-Injury vs. Post-Injury | 40312 | *< 0.0001 |
| 60:40 Model SOM Right CeA | Pre-Injury vs. Post-Injury | 21256 | *0.00916 |
| 30:70 Model PKC$\delta$ Left CeA | Pre-Injury vs. Post-Injury | 11819 | *0.00020 |
| 30:70 Model SOM Left CeA | Pre-Injury vs. Post-Injury | 56546 | *< 0.0001 |
| 30:70 Model PKC$\delta$ Right CeA | Pre-Injury vs. Post-Injury | 16268 | *< 0.0001 |
| 30:70 Model SOM Right CeA | Pre-Injury vs. Post-Injury | 46402 | *< 0.0001 |
| 50:50 Model PKC$\delta$ Pre-Injury | Left vs. Right | 44778 | 0.2754 |
| 50:50 Model PKC$\delta$ Post-Injury | Left vs. Right | 42888 | 0.9002 |
| 50:50 Model SOM Pre-Injury | Left vs. Right | 16494 | 0.7662 |
| 50:50 Model SOM Post-Injury | Left vs. Right | 36827 | 0.4121 |
| 60:40 Model PKC$\delta$ Pre-Injury | Left vs. Right | 51336 | 0.3599 |
| 60:40 Model PKC$\delta$ Post-Injury | Left vs. Right | 54325 | 0.7217 |
| 60:40 Model SOM Pre-Injury | Left vs. Right | 12706 | 0.7828 |
| 60:40 Model SOM Post-Injury | Left vs. Right | 27301 | 0.9783 |
| 30:70 Model PKC$\delta$ Pre-Injury | Left vs. Right | 19473 | 0.5907 |
| 30:70 Model PKC$\delta$ Post-Injury | Left vs. Right | 17630 | 0.2529 |
| 30:70 Model SOM Pre-Injury | Left vs. Right | 28951 | 0.6955 |
| 30:70 Model SOM Post-Injury | Left vs. Right | 63144 | 0.7148 |
| PKC$\delta$ Left CeA Pre-Injury | 50:50 vs. 60:40 | 45996 | 0.4147 |
| PKC$\delta$ Left CeA Post-Injury | 50:50 vs. 60:40 | 46780 | 0.6649 |
| SOM Left CeA Pre-Injury | 50:50 vs. 60:40 | 14534 | 0.7257 |
| SOM Left CeA Post-Injury | 50:50 vs. 60:40 | 30143 | 0.4965 |
| PKC$\delta$ Right CeA Pre-Injury | 50:50 vs. 60:40 | 50312 | 0.2296 |
| PKC$\delta$ Right CeA Post-Injury | 50:50 vs. 60:40 | 46328 | 0.5244 |
| SOM Right CeA Pre-Injury | 50:50 vs. 60:40 | 14584 | 0.6841 |
| SOM Right CeA Post-Injury | 50:50 vs. 60:40 | 30980 | 0.9465 |
| PKC$\delta$ Left CeA Pre-Injury | 50:50 vs. 30:70 | 25492 | 0.9664 |
| PKC$\delta$ Left CeA Post-Injury | 50:50 vs. 30:70 | 23768 | 0.207 |
| SOM Left CeA Pre-Injury | 50:50 vs. 30:70 | 23530 | 0.4603 |
| SOM Left CeA Post-Injury | 50:50 vs. 30:70 | 45515 | 0.08896 |
| PKC$\delta$ Right CeA Pre-Injury | 50:50 vs. 30:70 | 31883 | 0.825 |
| PKC$\delta$ Right CeA Post-Injury | 50:50 vs. 30:70 | 31675 | 0.9325 |
| SOM Right CeA Pre-Injury | 50:50 vs. 30:70 | 14584 | 0.6841 |
| SOM Right CeA Post-Injury | 50:50 vs. 30:70 | 30980 | 0.9465 |
| PKC$\delta$ Left CeA Pre-Injury | 60:40 vs. 30:70 | 29544 | 0.5309 |
| PKC$\delta$ Left CeA Post-Injury | 60:40 vs. 30:70 | 27084 | 0.3238 |
| SOM Left CeA Pre-Injury | 60:40 vs. 30:70 | 20010 | 0.8766 |
| SOM Left CeA Post-Injury | 60:40 vs. 30:70 | 41380 | 0.3580 |
| PKC$\delta$ Right CeA Pre-Injury | 60:40 vs. 30:70 | 33886 | 0.4062 |
| PKC$\delta$ Right CeA Post-Injury | 60:40 vs. 30:70 | 36418 | 0.5380 |
| SOM Right CeA Pre-Injury | 60:40 vs. 30:70 | 18012 | 0.8828 |
| SOM Right CeA Post-Injury | 60:40 vs. 30:70 | 36919 | 0.2463 |
