# Supplementary material for: Agent-based modeling of the central amygdala and pain using cell-type specific physiological parameters
Source: PLoS Comput Biol. 2021 Jun 8;17(6):e1009097. doi: 10.1371/journal.pcbi.1009097 (PMC8213159; doi:10.1371/journal.pcbi.1009097)
Supplement: S3 Table — Low values are indicative of mechanical hypersensitivity. Mean and SD of each group is given at baseline (before injection) and 1 hour after injection of the DREADD activator Clozapine N-oxide (CNO) or control saline (Sal). (DOCX) [file pcbi.1009097.s004.docx]

**S3 Table: Raw data from Wilson et al used for mean effect size calculations.** Low values are indicative of mechanical hypersensitivity. Mean and SD of each group is given at baseline (before injection) and 1 hour after injection of the DREADD activator Clozapine N-oxide (CNO) or control saline (Sal).

| **Number of animals in group** | **Strain** | **cuff/sham** | **Treatment** | **DREADD** | **Paw (injured side)** | **Before Injection Mean (g)** | **SD** | **1 hr after injection Mean (g)** | **SD** |
| --- | --- | --- | --- | --- | --- | --- | --- | --- | --- |
| 7 | PKC$\delta$ | Cuff | CNO | Gi | Left | 0.042 | 0.033 | 0.663 | 0.105 |
| 5 | PKC$\delta$ | Cuff | Sal | Gi | Left | 0.045 | 0.040 | 0.045 | 0.021 |
| 4 | PKC$\delta$ | Sham | CNO | Gi | Left | 0.810 | 0.225 | 0.770 | 0.050 |
| 2 | PKC$\delta$ | Sham | Sal | Gi | Left | 0.680 | 0.226 | 0.600 | 0.226 |
| 6 | SOM | Cuff | CNO | Gi | Left | 0.035 | 0.011 | 0.038 | 0.007 |
| 6 | SOM | Cuff | Sal | Gi | Left | 0.035 | 0.019 | 0.045 | 0.009 |
| 4 | SOM | Sham | CNO | Gi | Left | 0.408 | 0.053 | 0.030 | 0.012 |
| 4 | SOM | Sham | Sal | Gi | Left | 0.436 | 0.094 | 0.438 | 0.036 |
