# Supplementary material for: Agent-based modeling of the central amygdala and pain using cell-type specific physiological parameters
Source: PLoS Comput Biol. 2021 Jun 8;17(6):e1009097. doi: 10.1371/journal.pcbi.1009097 (PMC8213159; doi:10.1371/journal.pcbi.1009097)
Supplement: S4 Table — (DOCX) [file pcbi.1009097.s005.docx]

**S4 Table: Hedges’ *g* values calculated for *in vivo* studies and three models.**

| **PKC**$\delta$ **Manipulated** | | | | | | | | | | | | | | | | |
| --- | --- | --- | --- | --- | --- | --- | --- | --- | --- | --- | --- | --- | --- | --- | --- | --- |
|  | **Injured intact vs Control Intact** | | | | **Injured Inhibited vs Control Inhibited** | | | | **Control inhibited vs Control Intact** | | | | **Injured inhibited vs injured intact** | | | |
|  | **Wilson et al** | **30:70** | **50:50** | **60:40** | **Wilson et al** | **30:70** | **50:50** | **60:40** | **Wilson et al** | **30:70** | **50:50** | **60:40** | **Wilson et al** | **30:70** | **50:50** | **60:40** |
| **Hedges’ g** | 5.3 | 14.85 | 18.72 | 18.47 | 1.08 | -3.46 | -2.95 | -4.66 | 0.21 | -2.51 | -0.84 | -0.89 | -6.92 | -13.08 | -19.91 | -22.56 |
| **95% CI** | 2.86 | 8.47 | 10.73 | 10.58 | 2.4924 | -6.02 | -5.23 | -7.91 | 1.62 | -4.56 | -2.23 | -2.29 | -3.54 | -21.54 | -32.23 | -36.5 |
|  | 8.68 | 24.07 | 30.31 | 29.91 | -0.189 | -1.58 | -1.23 | -2.36 | -1.15 | -0.92 | 0.41 | 0.37 | -12.39 | -7.43 | -11.42 | -12.96 |
|  |  |  |  |  |  |  |  |  |  |  |  |  |  |  |  |  |
|  |  |  |  |  |  |  |  |  |  |  |  |  |  |  |  |  |
| **SOM Manipulated** | | | | | | | | | | | | | | | | |
|  | **Injured intact vs Control Intact** | | | | **Injured Inhibited vs Control Inhibited** | | | | **Control inhibited vs Control Intact** | | | | **Injured inhibited vs injured intact** | | | |
|  | **Wilson et al** | **30:70** | **50:50** | **60:40** | **Wilson et al** | **30:70** | **50:50** | **60:40** | **Wilson et al** | **30:70** | **50:50** | **60:40** | **Wilson et al** | **30:70** | **50:50** | **60:40** |
| **Hedges’ g** | 10.02 | 14.85 | 18.72 | 18.47 | 0.8 | 28.8 | 34.37 | 31 | 7.15 | 49.92 | 18.19 | 14.99 | -0.23 | 31.23 | 18.67 | 13.4 |
| **95% CI** | 5.62 | 8.47 | 10.73 | 10.58 | 2.19 | 16.58 | 19.82 | 17.86 | 2.54 | 26.43 | 10.42 | 8.55 | -0.66 | 17.99 | 10.7 | 7.62 |
|  | 16.56 | 24.07 | 30.31 | 29.91 | -0.47 | 46.56 | 55.55 | 50.1 | 17.42 | 80.65 | 29.45 | 24.3 | 0.13 | 50.48 | 30.23 | 22.06 |
