# Supplementary material for: Agent-based modeling of the central amygdala and pain using cell-type specific physiological parameters
Source: PLoS Comput Biol. 2021 Jun 8;17(6):e1009097. doi: 10.1371/journal.pcbi.1009097 (PMC8213159; doi:10.1371/journal.pcbi.1009097)
Supplement: S1 Text — (DOCX) [file pcbi.1009097.s006.docx]

**S1 Text: Instructions for Accessing and Simulating the Model**

Below are step-by-step instructions for downloading the NetLogo code and input files for the agent-based model. The Netlogo code and input files are located on the Open Science Framework public repository site <https://osf.io/nw5kx/> (doi: 10.0.68.197/OSF.IO/NW5KX).

**Instructions for downloading and opening the model:**

1. Navigate to <https://osf.io/nw5kx/> and locate the component titled ‘NetLogo code and supporting files’.
2. Download the NetLogo code “WholeAmygdala16.nlogo” and all stimulation history files, titled “stimulationhistory….csv” to a folder on your computer. The NetLogo code and all input files must be saved in the same folder.
3. Navigate to <https://ccl.northwestern.edu/netlogo/download.shtml> and download the newest version of NetLogo for a computer (PC or Mac).
4. Once downloaded, open NetLogo. Open the model by clicking “File”, “Open”, and then navigating to the folder in which the model and input files are saved.

**Instructions for simulating the model using the NetLogo GUI (S1 Fig):**

1. (Optional) You can adjust model parameters using the sliders on the interface. In particular, you can adjust the proportion of neurons that are SOM in the left and right hemispheres by adjusting the sliders labeled ‘Left-SOM’ and ‘Right-SOM’, respectively. You can adjust the proportions of SOM and PKCδ neurons that are RS and LF by adjusting the sliders labeled ‘Left-SOM-regular’, ‘Left-SOM-late’, ‘Left-PKC-regular’, and so on. You can adjust the firing rates of the spontaneous neurons using the sliders labeled “PKC-Spontaneous-Firing-Rate” and “SOM-Spontaneous-Firing-Rate”.
2. In the drop-down menu titled “File-To-Use”, choose the current stimulation history file you want to use. You may select from one of the files provided or create your own. Please see below for a description of the provided stimulation files.
3. Adjust the neural network switch to ‘On’ or ‘Off’ based on whether or not neurons are allowed to send inhibitory signals to one another each time step. If the switch is turned on, then you can use the corresponding sliders to modify the maximum number of incoming connections and outgoing connections each neuron may have. Additionally, you can adjust the inhibition threshold slider to indicate the minimum strength of an incoming signal needed to silence a neuron. If the switch is turned off, the network will not be created during initialization and neurons will not send inhibitory signals to one another during simulation.
4. After establishing all parameters and network settings, press the button labeled “Initialize”.
5. If you want to silence any neuron populations during the simulation, press the appropriate buttons labeled “Silent Left PKC neurons”, “Silent Left SOM neurons”, etc. These buttons can also be pressed during a simulation.
6. To start the simulation, press the Go button.
7. To pause the simulation, press the Go button again.
8. To adjust the speed of the simulation, use the built-in slider above the tick counter at the top of the interface.

To extract data from one or more model simulations, we recommend using NetLogo’s built-in tool called BehaviorSpace. To run BehaviorSpace with the model, chose the BehaviorSpace item from the Tools menu. The BehaviourSpace menu allows a user to specify the values of all switches and sliders on the user interface to be used in the simulation and the number of repetitions for each simulation. The menu also allows the user to specify the values to be reported during each simulation. In our simulations, we frequently reported the value of pain at each time step. BehaviourSpace then simulates the model according to the user’s specifications and outputs all reported values in a .csv file for further analysis.

**Description of stimulation history files provided with the model**

There are multiple stimulation history files (named “stimulationhistory….csv”) available for download with our NetLogo code at <https://osf.io/nw5kx/>. Below we provide a brief description of the stimulation simulated using the data in each file. The first two files below correspond to the constant 120 pA and ramping stimulation scenarios described in the paper. The other files were created for testing and calibrating the model.

1. stimulationhistory120.csv: This file assumes the stimulation is 120 pA for the entire simulation (300 timesteps). This is known as the ‘Constant 120 pA Scenario’.
2. stimulationhistory2.csv: This file assumes stimulation increases from 120 pA to 220 pA over first 330 time steps. Increases in stimulation occur in increments of 20 pA every 55 time steps. During the last 55 time steps (331 – 385), stimulation returns to 120 pA. This is known as the ‘Ramping Scenario’.
3. stimulationhistory0-20.csv: This file assumes the stimulation increases from 0 pA to 20 pA over 100 time steps. Increases in stimulation occur in increments of 5 pA every 20 time steps.
4. stimulationhistory20-40.csv: This file assumes the stimulation increases from 20 pA to 40 pA over 100 time steps. Increases in stimulation occur in increments of 5 pA every 20 time steps.
5. stimulationhistory40-60.csv: This file assumes the stimulation increases from 40 pA to 60 pA over 100 time steps. Increases in stimulation occur in increments of 5 pA every 20 time steps.
6. stimulationhistory60-80.csv: This file assumes the stimulation increases from 60 pA to 80 pA over 100 time steps. Increases in stimulation occur in increments of 5 pA every 20 time steps.
7. stimulationhistory80-100.csv: This file assumes the stimulation increases from 80 pA to 100 pA over 100 time steps. Increases in stimulation occur in increments of 5 pA every 20 time steps.
8. stimulationhistory100-120.csv: This file assumes the stimulation increases from 100 pA to 120 pA over 100 time steps. Increases in stimulation occur in increments of 5 pA every 20 time steps.
9. stimulationhistory120-140.csv: This file assumes the stimulation increases from 120 pA to 140 pA over 100 time steps. Increases in stimulation occur in increments of 5 pA every 20 time steps.
10. stimulationhistory140-160.csv: This file assumes the stimulation increases from 140 pA to 160 pA over 100 time steps. Increases in stimulation occur in increments of 5 pA every 20 time steps.
11. stimulationhistory160-180.csv: This file assumes the stimulation increases from 160 pA to 180 pA over 100 time steps. Increases in stimulation occur in increments of 5 pA every 20 time steps.
12. stimulationhistory180-200.csv: This file assumes the stimulation increases from 180 pA to 200 pA over 100 time steps. Increases in stimulation occur in increments of 5 pA every 20 time steps.
13. stimulationhistory200-220.csv: This file assumes the stimulation increases from 200 pA to 220 pA over 100 time steps. Increases in stimulation occur in increments of 5 pA every 20 time steps.
14. stimulationhistory220-240.csv: This file assumes the stimulation increases from 220 pA to 240 pA over 100 time steps. Increases in stimulation occur in increments of 5 pA every 20 time steps.
15. stimulationhistory120-220-120.csv: This file assumes the stimulation is at 120 pA for the first 200 time steps (1 - 200), 220 pA for the next 100 time steps (201 – 300), and 120 pA for the last 100 time steps (301 – 400).
16. stimulationhistory120-220-120-short50.csv: This file assumes the stimulation is at 120 pA for the first 50 time steps (1 – 50), 220 for the next 100 time steps (51 – 150), and 120 pA for the last 100 time steps (151 – 250).
17. stimulationhistory120-220-120-short60.csv: This file assumes the stimulation is at 120 pA for the first 60 time steps (1 – 60), 220 for the next 100 time steps (61 – 160), and 120 pA for the last 90 time steps (161 – 250).
18. stimulationhistory120-220-120-short70.csv: This file assumes the stimulation is at 120 pA for the first 70 time steps (1 – 70), 220 for the next 100 time steps (71 – 170), and 120 pA for the last 80 time steps (171 – 250).
19. stimulationhistory120-220-120-short75.csv: This file assumes the stimulation is at 120 pA for the first 75 time steps (1 – 75), 220 for the next 100 time steps (76 – 175), and 120 pA for the last 75 time steps (176 – 250).
20. stimulationhistory120-220-120-short80.csv: This file assumes the stimulation is at 120 pA for the first 80 time steps (1 – 80), 220 for the next 100 time steps (81 – 180), and 120 pA for the last 70 time steps (181 – 250).
21. stimulationhistory120-220-120-short90.csv: This file assumes the stimulation is at 120 pA for the first 90 time steps (1 – 90), 220 for the next 100 time steps (91 – 190), and 120 pA for the last 60 time steps (191 – 250).
22. stimulationhistory120-220-120-short100.csv: This file assumes the stimulation is at 120 pA for the first 100 time steps (1 – 100), 220 for the next 100 time steps (101 – 200), and 120 pA for the last 50 time steps (201 – 250).
23. stimulationhistory120-220-120-short125.csv: This file assumes the stimulation is at 120 pA for the first 125 time steps (1 – 125), 220 for the next 100 time steps (126 – 225), and 120 pA for the last 25 time steps (226 – 250).
